# Trained immunity is regulated by T cell-induced CD40-TRAF6 signaling

**DOI:** 10.1016/j.celrep.2024.114664

**Published:** 2024-08-22

**Authors:** Maaike M.E. Jacobs, Rianne J.F. Maas, Inge Jonkman, Yutaka Negishi, Willem Tielemans Zamora, Cansu Yanginlar, Julia van Heck, Vasiliki Matzaraki, Joost H.A. Martens, Marijke Baltissen, Michiel Vermeulen, Judit Morla-Folch, Anna Ranzenigo, William Wang, Martin Umali, Jordi Ochando, Johan van der Vlag, Luuk B. Hilbrands, Leo A.B. Joosten, Mihai G. Netea, Willem J.M. Mulder, Mandy M.T. van Leent, Musa M. Mhlanga, Abraham J.P. Teunissen, Nils Rother, Raphaël Duivenvoorden

**Affiliations:** 1 Department of Nephrology, Radboud University Medical Center, Nijmegen, the Netherlands; 2 BioMedical Engineering and Imaging Institute, Icahn School of Medicine at Mount Sinai, New York, NY, USA; 3 Cardiovascular Research Institute, Icahn School of Medicine at Mount Sinai, New York, NY, USA; 4 Department of Internal Medicine and Radboud Center for Infectious Diseases, Radboud University Medical Center, Nijmegen, the Netherlands; 5 Department of Cell Biology, Faculty of Science, Radboud University, Nijmegen, the Netherlands; 6 Department of Human Genetics, Radboud University Medical Center, Nijmegen, the Netherlands; 7 Department of Molecular Biology, Faculty of Science, Oncode Institute, Radboud University Nijmegen, Nijmegen, the Netherlands; 8 Department of Oncological Sciences, Icahn School of Medicine at Mount Sinai, New York, NY, USA; 9 Transplant Immunology Unit, National Center of Microbiology, Instituto de Salud Carlos III, Madrid, Spain; 10 Department of Medical Genetics, University of Medicine and Pharmacy, Iuliu Haƫieganu, Cluj-Napoca, Romania; 11 Department of Immunology and Metabolism, Life and Medical Sciences Institute, University of Bonn, Bonn, Germany; 12 Laboratory of Chemical Biology, Department of Biomedical Engineering and Institute for Complex Molecular Systems, Eindhoven University of Technology, Eindhoven, the Netherlands; 13 Department of Diagnostic, Molecular and Interventional Radiology, Icahn School of Medicine at Mount Sinai, New York, NY, USA; 14 Icahn Genomics Institute, Icahn School of Medicine at Mount Sinai, New York, NY, USA; 15 Lead contact

## Abstract

Trained immunity is characterized by histone modifications and metabolic changes in innate immune cells following exposure to inflammatory signals, leading to heightened responsiveness to secondary stimuli. Although our understanding of the molecular regulation of trained immunity has increased, the role of adaptive immune cells herein remains largely unknown. Here, we show that T cells modulate trained immunity via cluster of differentiation 40-tissue necrosis factor receptor-associated factor 6 (CD40-TRAF6) signaling. CD40-TRAF6 inhibition modulates functional, transcriptomic, and metabolic reprogramming and modifies histone 3 lysine 4 trimethylation associated with trained immunity. Besides *in vitro* studies, we reveal that single-nucleotide polymorphisms in the proximity of *CD40* are linked to trained immunity responses *in vivo* and that combining CD40-TRAF6 inhibition with cytotoxic T lymphocyte antigen 4-immunoglobulin (CTLA4-Ig)-mediated co-stimulatory blockade induces long-term graft acceptance in a murine heart transplantation model. Combined, our results reveal that trained immunity is modulated by CD40-TRAF6 signaling between myeloid and adaptive immune cells and that this can be leveraged for therapeutic purposes.

## In brief

Jacobs et al. show that T cells modulate trained immunity induction via CD40-TRAF6 signaling in monocytes. Blockade of CD40-TRAF6 signaling in monocytes inhibits trained immunity induction by affecting monocyte transcriptome, metabolism, and epigenome *in vitro* and prolongs allograft survival in a murine heart transplantation model.

## Graphical Abstract

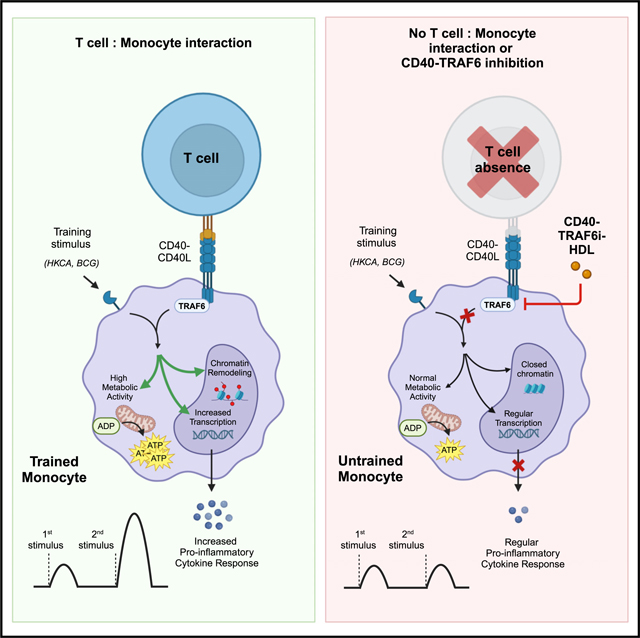

## INTRODUCTION

In the previous decade, the paradigm that immunological memory is a distinctive feature of the adaptive immune system has been refuted by studies revealing that innate immune cells can also develop immunological memory. This memory can occur in the stem cell niche of the bone marrow or in the periphery in cells that are circulating or residing in tissues.^1–3^ Innate immune memory, also termed trained immunity, involves metabolic and epigenetic changes induced by an initial stimulus. This stimulus can be certain microorganisms, of which *Candida albicans* and Bacillus Calmette-Guérin (BCG) are well-studied examples, or sterile inflammatory triggers, such as damage-associated molecular patterns (DAMPs) and inflammatory cytokines (e.g., interleukin-1β [IL-1β]).^4–7^ The resulting epigenetic changes facilitate the transcription of inflammatory genes that cause innate immune cells to develop a stronger inflammatory response upon restimulation.^1^ Metabolic rewiring is essential for mediating these epigenetic alterations by supplying metabolites serving as substrates for epigenetic enzymes or as co-activators or co-repressors for enzymes involved in writing or erasing epigenetic marks.^8^ By increasing inflammatory responsiveness, trained immunity provides a functional adaptation to protect the host from infection. However, trained immunity can also cause maladaptive immune responses in autoinflammatory disorders, atherosclerosis, cancer, and organ transplantation.^7,9–11^

Prior research has identified triggers capable of inducing trained immunity and elucidated intracellular signaling pathways that regulate the metabolic and epigenetic changes that underlie this phenomenon.^12,13^ However, little is known about how adaptive immune cells modulate trained immunity.^14,15^ As T cells play an essential role in modifying innate immune cell activation and differentiation, it is conceivable that T cells have a modulatory effect on trained immunity.^16^ Unraveling the role of the adaptive immune system in trained immunity is, therefore, imperative to better understand host defense mechanisms and gain new insights into the pathology of immune-mediated diseases.

In this study, we investigated the role of T cells in modifying trained immunity. By examining the interaction between T cells and monocytes *in vitro*, we found that T cells contribute to the training of monocytes. We show that this supportive role of T cells in trained immunity is mediated by cellular interactions between monocytes and T cells and involves cluster of differentiation 40 (CD40) activation on the monocyte. By combining transcriptomic, metabolomic, and epigenetic studies, we reveal that inhibiting CD40-tissue necrosis factor receptor-associated factor 6 (TRAF6) signaling attenuates trained immunity in monocytes. This important role for CD40-TRAF6 signaling was corroborated by the identification of single-nucleotide polymorphisms (SNPs) in the proximity of the *CD40* gene that were associated with altered trained immunity responses in humans. Next, we investigated the efficacy of modifying trained immunity by *in vivo* CD40-TRAF6 blockade. To this end, we used CD40-TRAF6 inhibitor-loaded nanobiologics (CD40-TRAF6i-NBs) with a high propensity for myeloid cell uptake. In a murine heart transplantation model, we found that short-term CD40-TRAF6i-NB therapy, combined with co-stimulatory blockade by cytotoxic T lymphocyte antigen 4-immunoglobulin (CTLA4-Ig), prolongs allograft survival. Our findings provide insight into how T cells shape trained immunity and identify CD40-TRAF6 signaling as an essential modulator.

## RESULTS

### T cells potentiate trained immunity in monocytes

**Figure 1.**
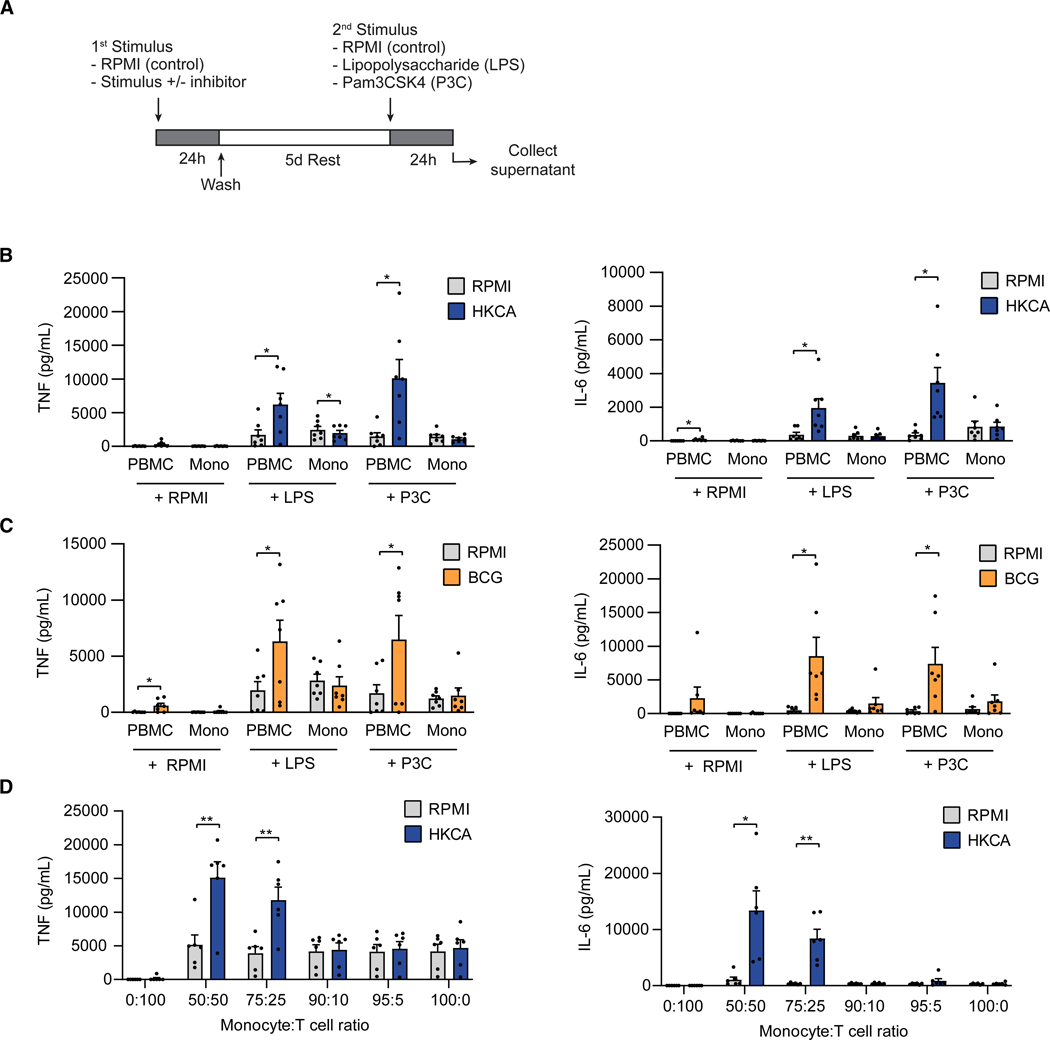
T cells modulate trained immunity responses in monocytes (A) Schematic representation of the *in vitro* assays for evaluating trained immunity induction. (B) TNF and IL-6 production in adherent PBMCs and purified monocytes not treated or treated with heat-killed *Candida albicans* (HKCA) upon RPMI, lipopolysaccharide (LPS), or Pam3CSK4 (P3C) restimulation (*n* = 7 donors). (C) TNF and IL-6 production in adherent PBMCs and purified monocytes not treated or treated with Bacille Calmette Guérin (BCG) vaccine after RPMI, LPS, or P3C restimulation (*n* = 7 donors). (D) TNF and IL-6 production in HKCA-treated or untreated autologous monocytes:T cells co-cultured at different ratios after restimulation with LPS (*n* = 6 donors). Mean ± SEM are shown, *p < 0.05 and **p < 0.01. Paired t tests were used. See also Figures S1 and S2.

We studied the role of T cells in trained immunity by comparing its induction in adherent peripheral blood mononuclear cells (PBMCs), which comprise around 50%–75% of T cells,^17^ to that in monocytes purified from healthy donor PBMCs by magnetic-assisted cell sorting (MACS). We used a previously described protocol to induce trained immunity *in vitro* using heat-killed *Candida albicans* (HKCA) or BCG vaccine, which are well-studied inducers of trained immunity.^18–20^ Adherent PBMCs and purified monocytes were stimulated for 24 h with HKCA, BCG vaccine, or RPMI culture medium (control), after which cells were rested for 5 days (Figure 1A). Monocyte differentiation in our assay was not different between HKCA-stimulated versus unstimulated (RPMI) PBMCs (Figure S1). After the resting period, cells were restimulated for 24 h with the Toll-like receptor (TLR)-4 agonist lipopolysaccharide (LPS) or the TLR-1/2 agonist Pam3Cys-Ser-(Lys)4 (Pam3CSK4), after which tumor necrosis factor (TNF) and IL-6 production was measured in the supernatant. Trained immunity was assessed by the difference in TNF and IL-6 production between untrained and trained cells.

HKCA-stimulated PBMCs showed increased TNF and IL-6 production after LPS or Pam3CSK4 restimulation compared to untrained cells (Figure 1B). In contrast, purified monocytes stimulated with HKCA showed no enhanced TNF or IL-6 production after restimulation, suggesting that T cells affect monocyte training. The marked difference between trained immunity induction in adherent PBMC cultures versus purified monocytes was also observed when cells were incubated with the BCG vaccine as a primary stimulus, indicating that this is not an HKCA-specific effect (Figure 1C). The augmented production of IL-6 and TNF observed in HKCA-induced trained immunity in adherent PBMCs did not result from cytokine production by adaptive immune cells, as confirmed by flow cytometry (Figure S2).

To further investigate the role of T cells in the observed difference in training capacity between adherent PBMCs and purified monocytes, we performed HKCA training experiments with co-cultures of autologous T cells and monocytes. We purified T cells (95%–99% purity) and monocytes (>90% purity) from PBMCs using MACS and co-cultured them for 7 days in different ratios using consistent numbers of monocytes per condition. We observed increased TNF and IL-6 production after LPS restimulation in HKCA-trained co-cultures with monocyte:T cell ratios of 50:50 and 75:25 (Figure 1D). This effect was, however, blunted in monocyte:T cell ratios of 90:10, 95:5, and 100:0, demonstrating that T cells potentiate HKCA-induced monocyte training.

### CD40-TRAF6 inhibition suppresses trained immunity responses

**Figure 2.**
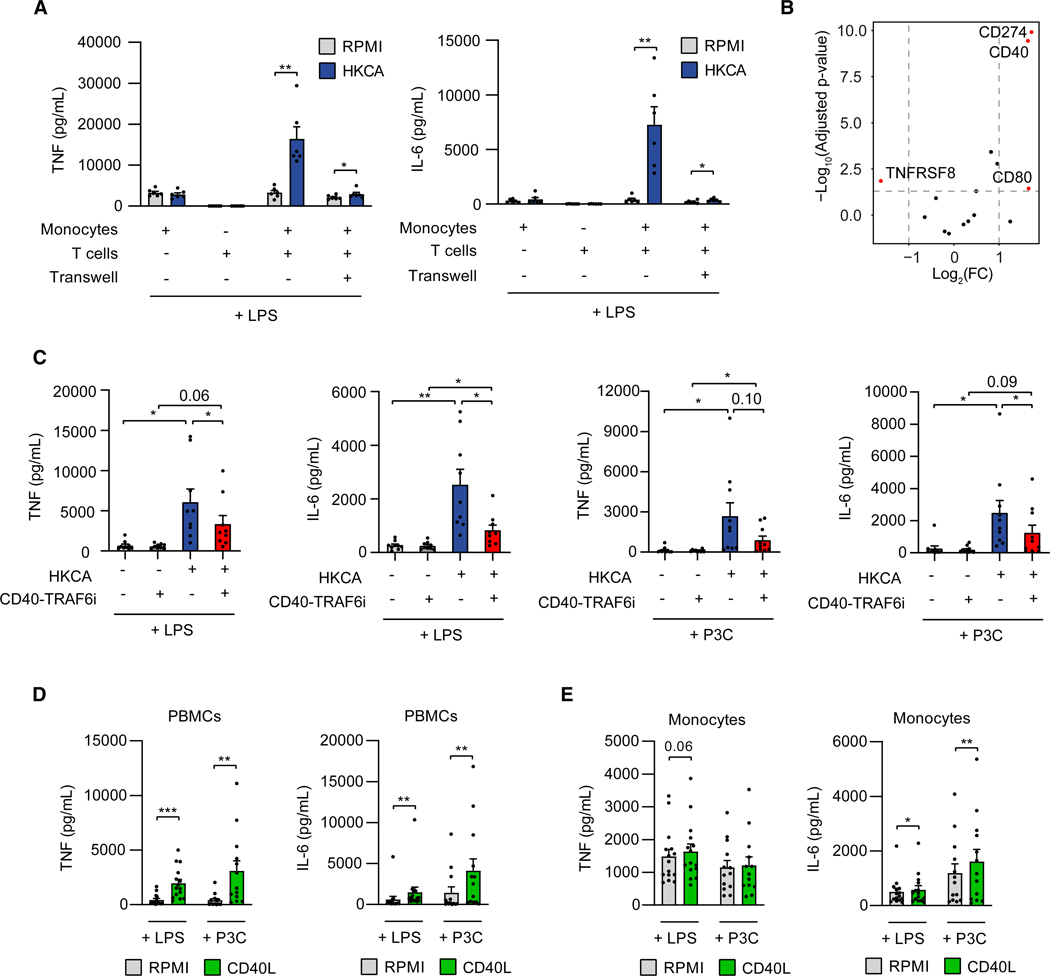
Trained immunity induction is mediated via CD40-TRAF6 signaling in monocytes (A) TNF and IL-6 production in autologous monocyte:T cell co-cultures and Transwell co-cultures not treated or treated with HKCA after restimulation with LPS (*n* = 6 donors). (B) Volcano plot indicating RNA expression of genes encoding co-stimulatory and co-inhibitory receptors in HKCA-stimulated adherent PBMCs versus unstimulated PBMCs 24 h post-stimulation. Fold changes (FCs) and *p* values were calculated for each gene using pairwise analysis with DESeq2 (*n* = 3 donors per group). Dotted lines indicate an FC of >2 or <0.5, and Bonferroni-adjusted *p* value < 0.05. (C) TNF and IL-6 production in HKCA-treated and untreated adherent PBMCs in the presence or absence of CD40-TRAF6i after restimulation with LPS (*n* = 9 donors) or P3C (*n* = 10 donors). (D) TNF and IL-6 production in CD40 ligand (CD40L)-treated and untreated adherent PBMCs after restimulation with LPS (*n* = 15 donors) or P3C (*n* = 13 donors). (E) TNF and IL-6 production in CD40L-treated and untreated monocytes after restimulation with LPS (*n* = 15 donors) or P3C (*n* = 13 donors). Mean ± SEM are shown, *p < 0.05, **p < 0.01, and ***p < 0.001. Paired t tests and paired two-way ANOVA with Šidák’s post-test were used. See also Figures S3–S6 and Table S1.

In light of our observation that T cells enhance monocyte training, we investigated whether their modulatory role is mediated by direct cellular contact or soluble factors. To address this, we studied trained immunity induction in monocyte:T cell co-cultures using a Transwell culture system, allowing interaction via soluble factors only. Monocytes were plated in the lower compartment and T cells in the upper compartment in a 50:50 ratio. Following stimulation with HKCA for 24 h and subsequent restimulation with LPS after a 5-day resting period, monocytes in physical contact with T cells showed markedly enhanced TNF and IL-6 production. In contrast, TNF and IL-6 production was only minimally enhanced when cells were cultured in a Transwell system, indicating the indispensable role of cellular contact between T cells and monocytes in facilitating HKCA-induced trained immunity (Figure 2A).

To elucidate the mechanism underlying the enhanced trained immunity conferred by cellular contact between T cells and monocytes, we explored the expression of co-signaling molecules known to regulate cellular activation upon direct interaction.^21^ We performed RNA sequencing on monocytes isolated from PBMCs 24 h after HKCA or RPMI stimulation. We observed upregulated expression of *CD274* (programmed death-ligand 1 [PD-L1]), *CD40*, and *CD80* in HKCA-stimulated monocytes, while *TNFRSF8* expression was downregulated (fold change [FC] > 2 or < 0.5, adjusted *p* value < 0.05) (Figure 2B; Table S1). Flow cytometric analysis revealed that PBMCs stimulated for 24 h with HKCA showed higher CD40 and CD86 expression on monocytes compared to RPMI stimulation. Interestingly, this was not the case when purified monocytes were stimulated with HKCA (Figure S3A). Based on these findings, we investigated the role of PD-L1/PD-1, CD40-CD40 ligand (CD40L), and CD80/86-CD28 signaling in trained immunity. Stimulation of adherent PBMCs for 24 h with HKCA in the presence of an antibody blocking PD-L1/PD-1 signaling did not affect TNF and IL-6 production after LPS or Pam3CSK4 restimulation 5 days later (Figures S3B and S3C). Similarly, treatment with CTLA4-Ig, which inhibits co-stimulatory CD80/86-CD28 signaling, had no effect on LPS- or Pam3CSK4-induced TNF and IL-6 production in HKCA-treated PBMCs (Figure S3D).

Next, we explored the involvement of CD40-CD40L signaling by stimulating adherent PBMCs with HKCA or RPMI for 24 h in the presence or absence of the small-molecule inhibitor (SMI) 6877002. SMI 6877002 specifically binds to the CD40 binding site for TRAF6 at its C-terminal tail but not the TRAF6 binding sites of IL-1R-associated kinases (IRAKs) or the binding sites of other TRAF molecules to the CD40 receptor.^22–24^ TRAF6 is an essential signal transducer for nuclear factor κB (NF-κB)-induced pro-inflammatory cytokine production in monocytes and macrophages after CD40 stimulation.^25^ CD40-TRAF6 inhibition reduced TNF and IL-6 production after LPS and Pam3CSK4 restimulation in HKCA-trained PBMCs without inducing cytotoxic effects (Figures 2C and S3B). We confirmed this finding in a separate experiment in which we used the same protocol but measured intracellular TNF and IL-6 by flow cytometry in CD11b^+^ cells (Figures S2A and S3E).

Because inhibition of CD40-TRAF6 signaling reduced trained immunity, we wondered whether CD40 stimulation could induce trained immunity. Therefore, we stimulated adherent PBMCs and purified monocytes with CD40L for 24 h and measured TNF and IL-6 production after LPS or Pam3CSK4 restimulation. In PBMCs, we observed increased TNF and IL-6 production after LPS and Pam3CSK4 restimulation (Figure 2D). We observed a similar effect in purified monocytes for IL-6 production. However, the effect size was considerably smaller compared to the effects observed in PBMCs (Figure 2E). Stimulation with both CD40L and HKCA did not augment trained immunity compared to HKCA stimulation alone but enhanced trained immunity compared to CD40L stimulation in adherent PBMCs upon LPS restimulation (Figure S4A). In monocytes, combining HKCA and CD40L stimulation was insufficient to induce HKCA-mediated training responses to the extent observed in PBMCs (Figure S4B). These data suggest that CD40 signaling contributes to trained immunity induction but that additional signals are required to elicit potent trained immunity responses.

As combined CD40 and interferon gamma (IFN-γ) stimulation is a classical innate immune cell stimulus, we investigated the potential contributory role of IFN-γ in trained immunity induction.^26^ In HKCA-stimulated monocyte:T cell co-cultures, IFN-γ production was limited 24 h post-stimulation, whereas a substantial increase in IFN-γ production was noted 6 days post-stimulation compared to RPMI-stimulated co-cultures (Figure S5A). Notably, HKCA stimulation of monocytes and T cells co-cultured in a Transwell system resulted in limited IFN-γ production at day 6 post-stimulation, suggesting that cellular contact is required for potent IFN-γ production (Figure S5A). The need for cellular contact was further supported by the observation that CD40-TRAF6 inhibition suppressed IFN-γ production 6 days after PBMC stimulation with HKCA (Figure S5B).

Based on these findings, we hypothesized that the suppressive effect of CD40-TRAF6 inhibition on trained immunity involves the modulation of IFN-γ-mediated effects on monocytes. Stimulation of adherent PBMCs or purified monocytes with IFN-γ for 24 h tended to increase TNF and IL-6 production upon LPS, but not Pam3CSK4, restimulation. Moreover, increased TNF responses were observed in combination with CD40L stimulation upon LPS or Pam3CSK4 restimulation in PBMCs and upon Pam3CSK4 restimulation in monocytes (Figures S5C and S5D). Prolonged exposure of adherent PBMCs and monocytes to IFN-γ for 6 days resulted in increased IL-6 production upon both LPS and Pam3CSK4 restimulation (Figures S6A and S6B). There was no effect on the TNF response (Figures S6A and S6B). To investigate this further, we stimulated PBMCs for 24 h with HKCA and the CD40-TRAF6 inhibitor, in combination with a 6-day supplementation of IFN-γ. We found that the suppressive effect of CD40-TRAF6 on trained immunity could be partly reversed by the addition of IFN-γ for the IL-6 response, though not for the TNF response (Figure S6C).

### CD40-TRAF6 inhibition modulates gene transcription profiles in trained immunity

**Figure 3.**
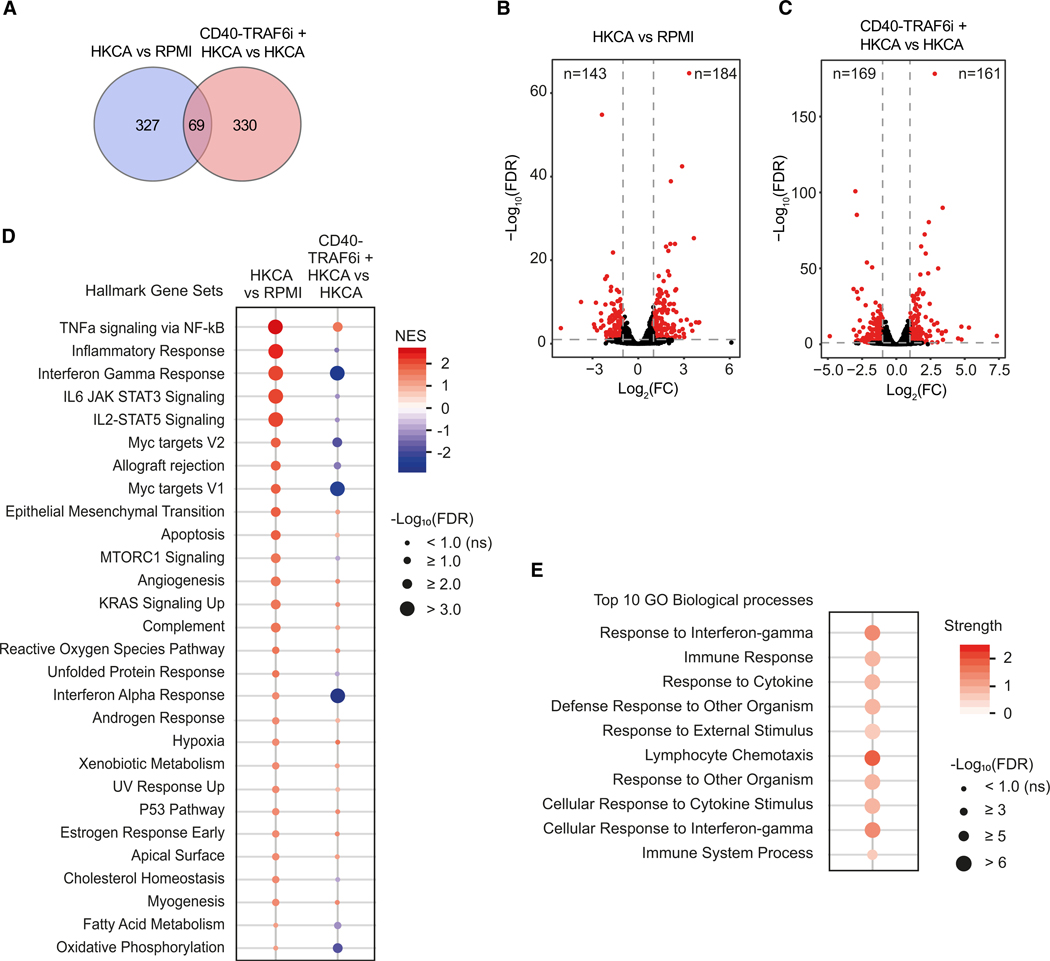
Effect of CD40-TRAF6 inhibition on transcriptional profiles in HKCA-trained monocytes (A) Number of differentially expressed genes (DEGs) in monocytes treated for 24 h with HKCA versus RPMI and HKCA plus CD40-TRAF6i versus HKCA only (FC > 2 or < 0.5, false discovery rate (FDR) < 0.1 (*n* = 3 donors per group). (B) Volcano plot indicating RNA expression in HKCA-stimulated monocytes versus unstimulated monocytes (RPMI) 24 h post-stimulation. FC and FDR were calculated for each gene using pairwise analysis with DESeq2 (*n* = 3 donors per group). Dotted lines indicate an FC of >2 or <0.5, and FDR < 0.1. (C) Volcano plot indicating RNA expression in HKCA-stimulated monocytes treated with CD40-TRAF6i versus HKCA-stimulated monocytes 24 h post-stimulation. FC and FDR were calculated for each gene using pairwise analysis with DESeq2 (*n* = 3 donors per group). Dotted lines indicate an FC of >2 or <0.5, and FDR < 0.1. (D) Significantly altered gene sets of the HALLMARK database in monocytes treated for 24 h with HKCA compared to RPMI or monocytes treated for 24 h with HKCA in the presence of CD40-TRAF6i compared to monocytes treated with HKCA alone (FDR < 0.1) (*n* = 3 donors per group). (E) Top 10 enriched Gene Ontology biological processes among 33 DEGs in HKCA-treated monocytes compared to RPMI-treated monocytes, for which expression is significantly reversed upon CD40-TRAF6i treatment, sorted on FDR (*n* = 3 donors per group). NES, normalized enrichment score. See also Figures S7 and S8.

To further dissect the molecular mechanisms by which CD40-TRAF6 inhibition allays the development of HKCA-induced trained immunity, we stimulated adherent PBMCs for 24 h with RPMI, HKCA, or HKCA with CD40-TRAF6 inhibition, after which we isolated monocytes from these PBMCs. We then performed RNA sequencing (RNA-seq) to inform us about the transcriptional changes that the training stimulus induces and that precede the development of the trained state. HKCA stimulation induced marked transcriptional changes compared with RPMI-treated cells, with 327 differentially expressed genes (DEGs; FC > 2 or < 0.5, false discovery rate [FDR] < 0.1). Inhibition of CD40-TRAF6 in HKCA-trained cells also caused marked transcriptional changes, with 330 DEGs compared to HKCA-trained cells (Figures 3A–3C). Gene set enrichment analysis using the HALLMARK database showed upregulation of pathways involved in immune responses in HKCA-stimulated monocytes compared with RPMI-treated cells. CD40-TRAF6 inhibition abrogated the effects of HKCA training on IFN-γ-stimulated genes, myelocytomatosis oncogene (MYC)-targeted genes, IFN-α-stimulated genes, and genes regulating oxidative phosphorylation (OXPHOS) (Figures 3D and S7A–S7E). The IFN-γ and IFN-α response pathways are largely mediated by the transcription factor signal transducer and activator of transcription, which induces the transcription of a large number of genes that play a central role in innate immune cell activation.^27^ MYC is a transcription factor that promotes the expression of numerous target genes that, among others, play a vital role in the metabolic reprogramming of innate immune cells.^28^ This indicates that in HKCA-induced trained immunity, CD40-TRAF6 signaling affects essential transcriptional programs related to inflammation and metabolic rewiring, which are critical features of trained macrophages.^29^

Interestingly, of the genes altered by HKCA, only a small fraction was modulated by CD40-TRAF6 inhibition. Of the 330 DEGs in HKCA-treated cells compared with RPMI, the expression of 33 genes was repressed, and 36 were upregulated by CD40-TRAF6 inhibition (Figures 3A, S8A, and S8B). When looking specifically at the 33 genes for which expression was reversed by CD40-TRAF6 inhibition, we found that these were mainly related to Gene Ontology terms relating to IFN-γ responses, external and cytokine stimuli, and chemotaxis (Figure 3E). The finding that a minor fraction of the HKCA-induced genes was altered by blocking CD40-TRAF6 signaling suggests that this pathway’s effect on trained immunity arises partly from reversing HKCA-induced gene expression but might also be attributable to other pathways regulated by CD40-TRAF6. The 261 genes not affected by HKCA training but whose expression was altered by CD40-TRAF6 inhibition were enriched in genes related to immune responses (Figure S8C).

### CD40-TRAF6 inhibition affects trained immunity-associated metabolic reprogramming

**Figure 4.**
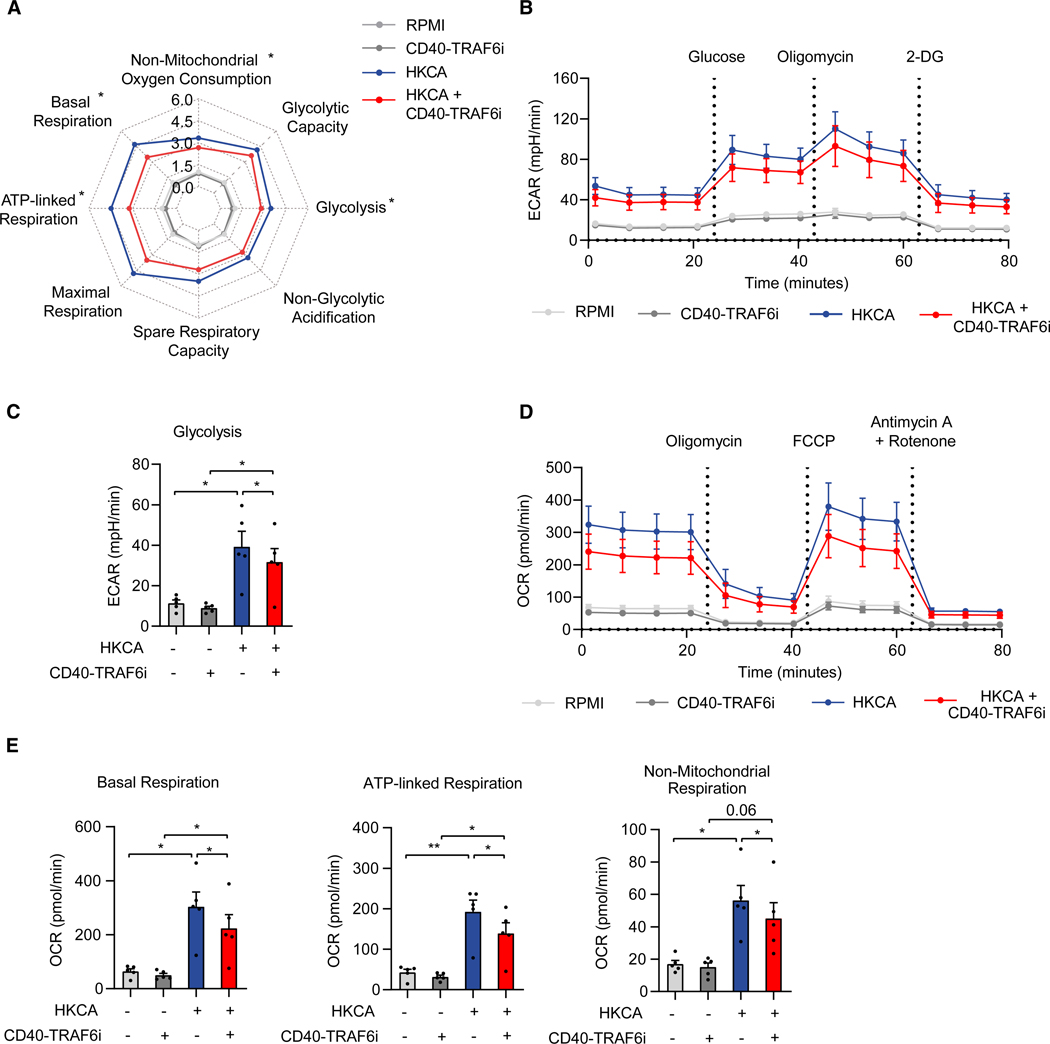
CD40-TRAF6 inhibition alters oxidative phosphorylation and glycolysis in HKCA-trained PBMCs (A) Spider plot showing metabolic parameters 6 days after stimulation in PBMCs that were not treated or treated with HKCA in the presence or absence of CD40-TRAF6i for 24 h (*n* = 5 donors). Values were normalized to untrained PBMCs. The asterisk (*) indicates a significant difference between HKCA+CD40-TRAF6i versus HKCA-treated PBMCs. (B) Extracellular acidification rate (ECAR) upon injection of glucose, oligomycin, and 2-deoxyglucose (2-DG) at indicated time points in PBMCs not treated or treated with HKCA in the presence or absence of CD40-TRAF6i, measured 6 days after treatment using Seahorse technology (*n* = 5 donors). (C) Glycolysis rate analyzed with Seahorse technology in PBMCs not treated or treated with HKCA in the presence or absence of CD40-TRAF6i for 24 h, 6 days after treatment (*n* = 5 donors). (D) Oxygen consumption rate (OCR) upon injection of oligomycin, carbonyl cyanide-4-(trifluoromethoxy)phenylhydrazone) (FCCP), and antimycin A + rotenone at indicated time points in PBMCs not treated or treated with HKCA in the presence or absence of CD40-TRAF6i, measured 6 days after treatment using Seahorse technology (*n* = 5 donors). (E) Basal respiration, ATP-linked respiration, and non-mitochondrial respiration in PBMCs not treated or HKCA-treated in the presence or absence of CD40-TRAF6i for 24 h, 6 days after treatment (*n* = 5 donors). Mean ± SEM are shown. *p < 0.05 and **p < 0.01. Paired t tests and paired two-way ANOVA with Šidák’s post-test were used. See also Figure S9.

Trained immunity induction is characterized by a marked upregulation of aerobic glycolysis and OXPHOS.^12^ We investigated the effect of CD40-TRAF6 inhibition on cellular metabolism by performing extracellular flux analysis. Adherent PBMCs were trained with HKCA or RPMI for 24 h in the presence or absence of CD40-TRAF6i as described before, and after the 5-day resting period, PBMCs were harvested for performing extracellular flux assays. Glycolytic parameters were assessed by measuring changes in the extracellular acidification rate in response to injections of glucose, oligomycin, and 2-deoxyglucose. OXPHOS parameters were assessed by measuring changes in the oxygen consumption rate in response to oligomycin, carbonyl cyanide 4-(trifluoromethoxy)phenylhydrazone, and rotenone/antimycin A. We found that CD40-TRAF6 inhibition upon HKCA training reduced glycolysis and the OXPHOS parameters basal respiration, ATP-linked respiration, and non-mitochondrial respiration, indicating that CD40-TRAF6 inhibition reduces the upregulation of metabolic processes underlying HKCA-induced trained immunity induction (Figures 4A–4E). We confirmed that CD40-TRAF6i specifically affects cell metabolism in monocytes by performing extracellular flux assays in monocytes purified from adherent PBMCs 5 days after the HKCA training stimulus (Figures S9A–S9C).

### CD40-TRAF6 inhibition affects the epigenetic regulation of trained immunity

**Figure 5.**
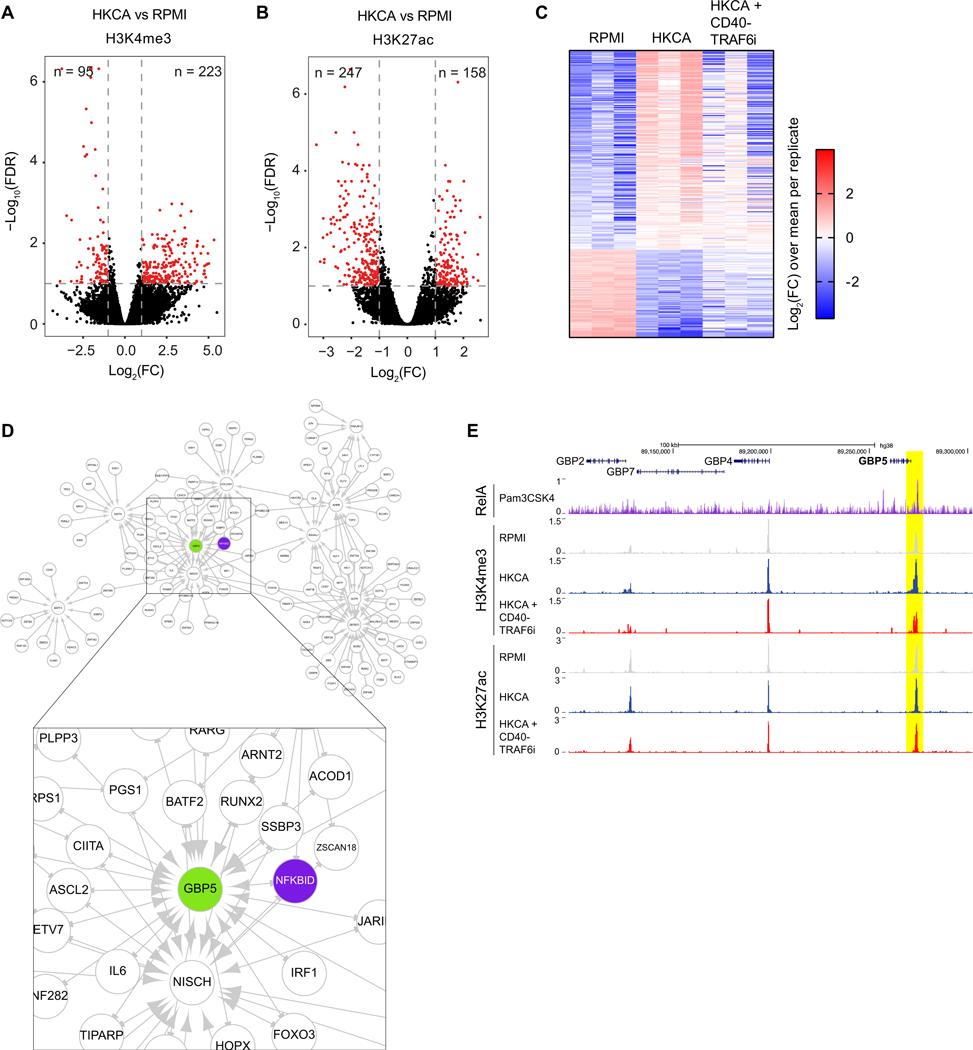
Effect of CD40-TRAF6 inhibition on H3K4me3 modifications of HKCA-trained monocytes (A) Volcano plot showing genomic regions with significantly altered H3K4me3 peak intensity (FC > 2 or < 0.5, FDR < 0.1) in HKCA-stimulated versus unstimulated monocytes (RPMI) 5 days post-stimulation (*n* = 3 donors), analyzed with DESeq2. (B) Volcano plot showing genomic regions with significantly altered H3K27ac peak intensity (FC > 2 or < 0.5, FDR < 0.1) in HKCA-stimulated versus unstimulated monocytes (RPMI) 6 days post-stimulation (*n* = 3 donors), analyzed with DESeq2. (C) Heatmap showing the intensity of H3K4me3 peaks in unstimulated monocytes (RPMI) and monocytes stimulated with HKCA in the presence or absence of CD40-TRAF6 inhibitor, for 318 genomic regions with significantly altered H3K4me3 peak intensity in HKCA-stimulated versus RPMI-stimulated monocytes (FC > 2 or < 0.5, FDR < 0.1) (*n* = 3 donors). (D) Gene regulatory network inferred from bulk RNA-seq showing genes where HKCA-induced expression was diminished by CD40-TRAF6 inhibitor. Boxed image on the bottom zooms in at NFKBID-GBP5. (E) Genomic view around GBP5. Tracks indicates RelA binding in P3C-treated THP-1 cells (available from ENCODE3), H3K4me3 and H3K27ac binding in monocytes treated with RPMI, or HKCA in the presence or absence of CD40-TRAF6 inhibitor. The yellow highlighted region is the GBP5 promoter. See also Figure S10.

Epigenetic changes underlie the transcriptional regulation of inflammatory genes in trained immunity.^12^ Therefore, we investigated the role of CD40-TRAF6 signaling in the epigenetic regulation of monocytes. To this end, we performed a genome-wide assessment of histone 3 lysine 4 trimethylation (H3K4me3) and histone 3 lysine 27 acetylation (H3K27ac) by chromatin immunoprecipitation sequencing (ChIP-seq). Adherent PBMCs were trained as previously described, and after 5 days of rest, monocytes were harvested from these PBMCs for ChIP-seq. ChIP-seq of H3K4me3 and H3K27ac revealed clear differences between HKCA-trained and untrained cells. We found 318 and 405 peaks showing a significant change in H3K4me3 and H3K27ac dynamics between HKCA and control cells, respectively (FC > 2 or < 0.5, FDR < 0.1) (Figures 5A and 5B). Pathway analysis of differentially regulated H3K4me3 peaks in HKCA-trained cells using the Genomic Regions Enrichment of Annotations Tool showed pathways associated with immune responses (Figure S10A). CD40-TRAF6 inhibition significantly reversed 46 of the 318 differentially regulated H3K4me3 peaks induced by HKCA training (Figures 5C and S10B). Analysis of the H3K27ac peaks differentially regulated between HKCA- and RPMI-treated cells showed pathways associated with the regulation of apoptosis and ATP metabolic processes (Figure S10A). CD40-TRAF6 inhibition had little effect on these H3K27ac peaks, significantly altering only eight of the 405 differentially regulated peaks between HKCA- and RPMI-treated cells (Figure S10C). These data corroborate our functional and metabolic analyses, demonstrating that CD40-TRAF6 inhibition influences histone modifications associated with trained immunity, particularly evident in changes related to H3k4me3.

To elucidate the mechanisms by which CD40-TRAF6 inhibition mediates these epigenetic changes, we conducted a gene regulatory network analysis on our RNA-seq results (Figure 3) using GENIE3. We observed that genes affected by CD40-TRAF6 inhibition, including GBP5, are controlled by multiple transcription factors (Figure 5D). GBP5 acts as an activator of NLRP3 inflammasome assembly and plays a crucial role in the innate immune response.^30^ The transcription factors regulating GBP5, namely NFKBID and BATF2, are known to interact with and modulate the activity of NF-κB and AP-1, respectively.^31,32^ RelA, a component of NF-κB, binds to the GBP5 promoter.^33^ We observed that the HKCA-induced increase in H3k4me3 intensity at the GBP5 promotor was reduced upon CD40-TRAF6 inhibition (Figure 5E).

### SNPs around the CD40 gene associate with trained immunity responses

To confirm a role for CD40 signaling in modulating trained immunity responses, we conducted functional trained immunity quantitative trait locus (FTI-QTL) analyses using genotype data and cytokine measurements of human PBMCs of 267 healthy participants in the 300BCG cohort of the Human Functional Genomics Project.^34^ This cohort was generated for a previous study investigating the impact of genetic variation on *in*-*vitro*-induced trained immunity by β-glucan and *in*-*vivo*-induced trained immunity by BCG vaccination and was comprised of adults in the age range of 18–71 years, of which 56% were female.^35^ Genotyping of DNA samples was performed to identify SNPs. Two different FTI-QTL analyses were performed, the first involving *ex vivo* training of PBMCs with BCG or β-glucan and the second with *in vivo* BCG vaccination as a training stimulus.

**Figure 6.**
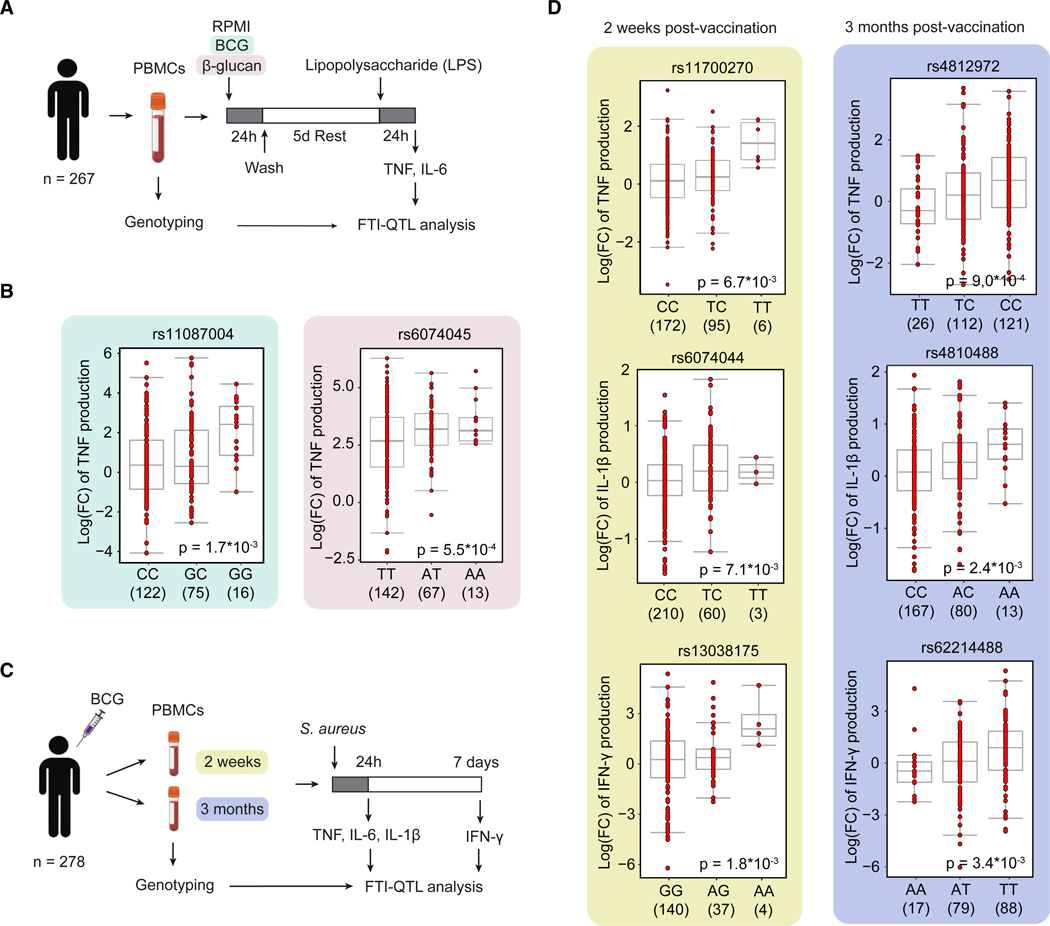
SNPs in the proximity of *CD40* associate with *ex vivo* and *in vivo* trained immunity responses (A) Experimental design for the FTI-QTL analysis with *in vitro* stimulation. (B) Associations of SNPs rs11087004 (*n* = 213, β = −0.58 [C versus G], *p* = 1.7 × 10^−3^) and rs6074045 (*n* = 222, β = −0.54 [T versus A], *p* = 5.5 × 10^−4^) in the proximity of *CD40* with TNF production for BCG- and β-glucan-induced trained immunity, respectively. Boxplots show genotype-stratified FCs of stimulated versus unstimulated cells. β-Values indicate effect size and direction. (C) Experimental design for the FTI-QTL analysis with *in vivo* BCG vaccination. (D) Associations of SNPs in *CD40* rs11700270 (*n* = 273, β = 0.32 [T versus C], *p* = 6.7 × 10^−3^), rs6074044 (*n* = 273, β = 0.21 [T versus C], *p* = 7.1 × 10^−3^), and rs13038175 (*n* = 181, β = −0.68 [G versus A], *p* = 1.8 × 10^−3^) with cytokine production of PBMCs collected 2 weeks after trained immunity induction with BCG vaccination, and SNPs in the proximity of *CD40* rs4812972 (*n* = 259, β = 0.34 [C versus T], *p* = 9.0 × 10^−4^), rs4810488 (*n* = 260, β = 0.20 [A versus C], *p* = 2.4 × 10^−3^), and rs62214488 (*n* = 184, β = −0.41 [A versus T], *p* = 3.4 × 10^−3^) with cytokine production of PBMCs collected 3 months after trained immunity induction using BCG vaccination, upon stimulation with *Staphylococcus aureus* (*S. aureus*). Boxplots show genotype-stratified FCs of cytokine production compared to PBMCs collected from the same individual before BCG vaccination. β-Values indicate effect size and direction. Production of TNF and IL-1β was measured 24 h after *S. aureus* stimulation, and production of IFN-γ was quantified 7 days after *S. aureus* stimulation.

In the initial FTI-QTL analysis, adherent PBMCs were stimulated *ex vivo* with RPMI (control), BCG, or β-glucan for 24 h to induce trained immunity, followed by quantification of TNF and IL-6 upon LPS restimulation 5 days later, as previously described by Moorlag et al. (Figure 6A).^35^ The analysis identified two SNPs in the proximity of the *CD40* gene (within a 250-kb window) that were associated with BCG- and β-glucan-induced trained immunity responses compared to unstimulated controls (Figure 6B). SNPs rs11087004 and rs6074045 were linked to the TNF response in BCG- (*n* = 213, β = −0.58 [C versus G], *p* = 1.7 × 10^−3^) and β-glucan-trained cells (*n* = 222, β = −0.54 [T versus A], *p* = 5.5 × 10^−4^), respectively (Figure 6B). No associations between SNPs in the proximity of the *CD40* gene and IL-6 responses were found.

For the second FTI-QTL analysis, BCG vaccination was performed in 278 participants of the 300BCG cohort, constituting an *in vivo* training stimulus. PBMCs were collected before vaccination and at 2 weeks and 3 months post-vaccination. Adherent PBMCs were *ex vivo* stimulated with *Staphylococcus aureus* (*S. aureus)* for 24 h, and TNF, IL-6, and IL-1β levels were measured in the supernatant.^36^ The difference in cytokine production before vaccination, compared to 2 weeks or 3 months after vaccination, quantified the trained immune response per subject (Figure 6C). In PBMCs collected 2 weeks post-vaccination, two SNPs in the proximity of the *CD40* gene (within a 250-kb window) were found to associate with trained immunity responses. rs11700270 (*n* = 273, β = 0.32 [T versus C], *p* = 6.7 × 10^−3^) was associated with the TNF response, and rs6074044 (*n* = 273, β = 0.21 [T versus C], *p* = 7.1 × 10^−3^) was associated with the IL-1β response. In PBMCs collected 3 months post-vaccination, rs4812972 (*n* = 259, β = 0.34 [T versus C], *p* = 9.0 × 10^−4^) was associated with the TNF response, and rs4810488 (*n* = 260, β = 0.20 [A versus C], *p* = 2.4 × 10^−3^) was associated with the IL-1β response (Figure 6D). No SNPs were associated with IL-6 responses of PBMCs collected 2 weeks or 3 months after BCG vaccination. In an additional analysis, PBMCs of BCG-vaccinated participants were *ex vivo* stimulated with *S. aureus* for 7 days, and IFN-γ levels were measured in the supernatant (Figure 6C). Two weeks post-vaccination, the SNP rs13038175 (*n* = 181, β = −0.68 [A versus G], *p* = 1.8 × 10^−3^), and 3 months post-vaccination, the SNP rs62214488 (*n* = 184, β = −0.41 [A versus T], *p* = 3.4 × 10^−3^), was associated with the IFN-γ response (Figure 6D).

### Inhibition of trained immunity and T cell co-stimulation promotes allograft acceptance

To exploit our finding that CD40-TRAF6 signaling plays a central role in potentiating trained immunity induction *in vivo*, we investigated the effect of CD40-TRAF6 inhibition in a heterotopic heart transplantation model. The immune system responds vigorously to alloantigens, and this involves a complex interaction between myeloid cells and adaptive immune cells.^37^ A previous study demonstrated a pathological role for trained immunity in the alloimmune response.^11^ Building on this, we conceived that inhibiting trained immunity might suppress the production of innate immune cell-derived cytokines (signal 3) that influence T cell differentiation. Specifically, we hypothesized that allograft tolerance can be promoted by attenuating trained-immunity-mediated pro-inflammatory cytokine production (signal 3) using CD40-TRAF6 inhibition alone and in combination with controlling active immune activation by T cell co-stimulatory blockade (signal 2) (Figure S11A). T cell co-stimulation can be inhibited with CTLA4-Ig, which is approved for treating organ transplant recipients.^38^

**Figure 7.**
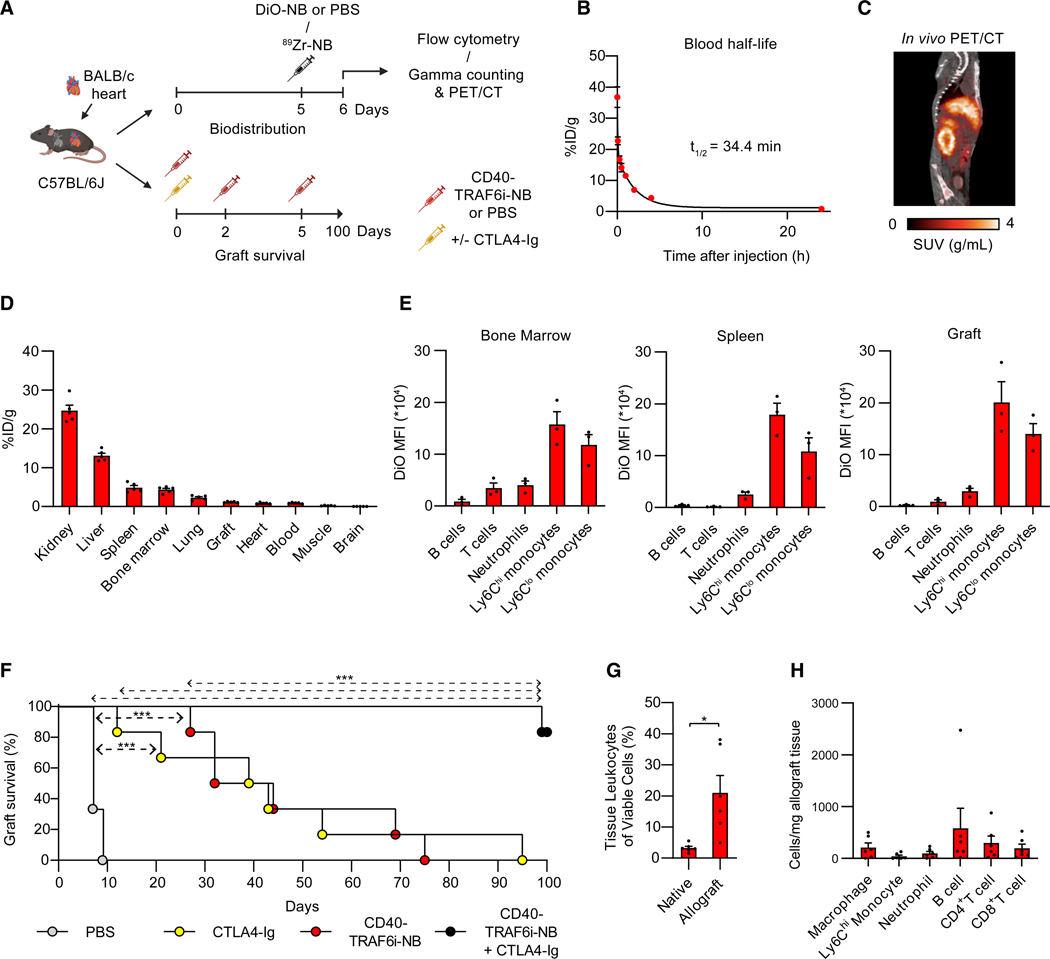
Myeloid-specific CD40-TRAF6 inhibition combined with CTLA4-Ig prolongs allograft survival in a heart transplant mouse model (A) Schematic of the experimental setup. (B) Determination of blood half-life time of ^89^Zr-NB (*n* = 5 mice/group). (C) Representative whole-body 2D positron emission tomography with computed tomography (PET/CT) image of a C57BL/6J mouse heterotopically transplanted with a BALB/c heart 24 h after injection of ^89^Zr-NBs. (D) Gamma counting of organs from C57BL/6J mice heterotopically transplanted with a BALB/c heart 24 h after ^89^Zr-NBs injection (*n* = 5 mice/group). (E) Flow cytometry of DiO-NB uptake in bone marrow, spleen, and graft 24 h after DiO-NB administration (*n* = 3 mice/group). (F) Kaplan-Meier curve with log-rank test for graft survival (*n* = 6 mice/group). ***p < 0.001. (G) Leukocytes infiltrated in native hearts and allografts of CTLA4-Ig plus CD40-TRAF6i-NB-treated mice 100 days post-transplantation (*n* = 6 mice). *p < 0.05, paired t test. (H) Immune cell subsets in allografts of CTLA4-Ig plus CD40-TRAF6i-NB-treated recipient mice 100 days post-transplantation (*n* = 6 mice). Mean ± SEM are shown. See also Figures S11 and S12.

Prior to assessing the effect of CD40-TRAF6 inhibition and CTLA4-Ig treatment on graft survival *in vivo*, CD40-TRAF6i was incorporated in apolipoprotein A1-based NBs that were previously shown to efficiently facilitate drug delivery to myeloid cells but not adaptive immune cells.^24^ Comparable to previous studies, the CD40-TRAF6i-NBs had a drug encapsulation efficiency of >90%, as determined by UV-visible spectrophotometry, and a mean hydrodynamic diameter of 22 nm (based on number distribution) and a dispersity of 0.26, determined by dynamic light scattering.^24^ To evaluate the blood clearance and biodistribution of NBs in a heterotopic heart transplantation model, CD40-TRAF6i-NBs were labeled with ^89^Zr (^89^Zr-NBs) and injected into C57BL/6J mice heterotopically transplanted with BALB/c hearts (Figure 7A). Blood clearance measurements showed a weighted blood half-life of 34.4 min for ^89^Zr-NBs (Figure 7B). Subsequently, we evaluated the biodistribution of ^89^Zr-NBs using a combination of *in vivo* positron emission tomography with computed tomography imaging, followed by *ex vivo* quantification of radioactive signal in murine tissues. ^89^Zr-NBs accumulated in kidney and liver, followed by spleen and bone marrow, 24 h post-injection of ^89^Zr-NBs (Figures 7C and 7D).

To confirm myeloid-specific uptake of CD40-TRAF6i-NBs, NBs were labeled with fluorescent DiO (DiO-NBs) and injected into C57BL/6J mice heterotopically transplanted with BALB/c hearts (Figure 7A). Bone marrow, spleens, and grafts were collected 24 h after injection, and the DiO signal in leukocyte subsets was assessed by flow cytometry. As expected, NBs were mainly taken up by myeloid cells, with monocytes in particular having high DiO uptake, whereas lymphocytes showed virtually no uptake (Figures 7E and S11B).

Next, we investigated the effect of CD40-TRAF6 inhibition and CTLA4-Ig treatment on graft survival of fully allogeneic donor hearts in a heterotopic heart transplantation mouse model in which C57BL/6J mice received a heart from BALB/c mice (Figure 7A). Recipient mice were treated with a single intraperitoneal dose of CTLA4-Ig (0.25 mg) on the day of transplantation, three intravenous injections of CD40-TRAF6i-NB (5 mg/kg) on the day of transplantation and post-operatively on days 2 and 5, or a combination of both therapies. Unloaded NBs (not containing drug) do not affect allograft survival.^11^ Treatment with CD40-TRAF6i-NBs significantly prolonged transplant survival compared with PBS-treated controls, which was similar to the effect of CTLA4-Ig therapy (Figure 7F). CD40-TRAF6i-NBs and CTLA4-Ig co-therapy showed a strong synergistic effect, resulting in a 99-day allograft survival of one allograft and >100-day survival of the remaining five allografts (Figure 7F). We subsequently performed flow cytometry on the native hearts, the five non-rejected allografts, and the rejected allograft of the mice treated with our co-therapy 100 days post-transplantation (Figures 7G and S12). As expected, the number of leukocytes in the allografts was higher than in native hearts, with a considerable variation in leukocyte numbers (Figure 7G). The graft-infiltrating leukocytes mainly consisted of macrophages (CD45^+^ CD11b^+^Ly6G^−^F4/80^+^), B cells (CD45^+^CD19^+^), and CD4^+^ and CD8^+^ T cells (CD45^+^CD3^+^CD4^+^ or CD45^+^CD3^+^CD8^+^) (Figure 7H).

## DISCUSSION

Our study identifies a modulatory role for T cells in trained immunity. We found that cellular contact between monocytes and T cells via CD40 signaling potentiates HKCA-induced trained immunity. We also demonstrated that inhibiting TRAF6 signaling downstream of CD40 activation facilitates modulation of the transcriptomic, metabolic, and epigenetic alterations underlying trained immunity. Using an FTI-QTL analysis, we showed associations between SNPs near the *CD40* gene and the cytokine responses associated with BCG- and β-glucan-induced trained immunity in healthy adults included in the Human Functional Genomics Project. Next, we applied the concept of inhibiting trained immunity via CD40-TRAF6 inhibition to a murine heterotopic heart transplantation model using myeloid-targeting NBs loaded with a CD40-TRAF6 inhibitor. We revealed that myeloid-specific inhibition of CD40-TRAF6 signaling, combined with co-stimulatory blockade with CTLA4-Ig, is an effective strategy for promoting allograft acceptance.

Here, we challenge the paradigm that trained immunity is an autonomous process in innate immune cells that is independent from the adaptive immune system by showing that T cells play a modifying role in the development of trained immunity responses in monocytes. Our data show that the ratio of monocytes to T cells dictates the training of monocytes by HKCA. Previous studies similarly found signs of a modifying role for T cells in trained immunity. In a study by Zhang et al., trained monocytes were analyzed by single-cell RNA-seq (scRNA-seq), and the results were compared with scRNA-seq data of trained PBMCs. The authors noticed that the presence of lymphocytes during monocyte training influenced the transcriptional signatures induced in these monocytes, showing increased expression of genes encoding for chemokines when lymphocytes were present.^15^ Yao et al. demonstrated that CD8^+^ T cells promote trained immunity induction in tissue-resident alveolar macrophages and that this relies on the production of IFN-γ by CD8^+^ T cells.^39^ In another study, Crabtree et al. showed that T cells contribute to trained immunity in human monocytes in response to *P. falciparum*-infected red blood cells via soluble factors, in particular IFN-γ.^40^ In contrast, our study shows that the induction of trained immunity requires direct interactions between T cells and monocytes, including CD40^−^CD40L interaction. Interestingly, we found that CD40-TRAF6 inhibition reduced IFN-γ production in PBMCs and that this reduced IFN-γ production partly contributed to suppression of trained immunity. This suggests that direct monocyte-T cell interactions induce IFN-γ production by T cells, which subsequently primes monocytes for increased responsiveness toward secondary stimuli. This concept is supported by the studies of Lee et al. and Tran et al., who highlight an important role for T cell-derived IFN-γ as a priming stimulus in mediating trained immunity induction by BCG.^41,42^ Furthermore, IFN-γ is known to enhance CD40 expression on myeloid cells, thereby potentiating CD40 signaling.^43,44^

In our *in vitro* experimental setup, we could not detect trained immunity responses at monocyte-to-T cell ratios of 90:10 and higher. However, the absence of such responses in this particular setup does not negate the potential of monocytes to undergo training in the absence or scarcity of T cells, as demonstrated in prior studies. Trained immunity constitutes an intrinsic process within innate immune cells, as evidenced by previous investigations using RAG-1-deficient and severe combined immunodeficiency mice, wherein the absence of T and B cells did not preclude the induction of trained immunity *in vivo.*^4,45,46^ Moreover, several studies described the induction of trained immunity in purified human monocytes under *in vitro* conditions, although the magnitude of these trained immune responses is generally lower than what is seen in adherent PBMCs.^4,18,47^ The varying purity of monocyte enrichment achieved using different isolation techniques, i.e., Percoll isolation or MACS-mediated monocyte isolation, likely contribute to the observed differences in trained immunity responses throughout different studies.^48–51^

We found a consistent role of CD40 signaling in trained immunity and the epigenetic and metabolic changes underlying it. Downstream CD40 signaling is mediated by the binding of multiple TRAF proteins (TRAF2/3/5 and −6) that interact with the two cytoplasmic binding domains of CD40.^52,53^ One binding domain is specific for TRAF6, while the other TRAFs share the other binding site. The interaction of the different TRAFs with the two binding sites has different functionalities.^52,53^ Notably, the interaction between CD40 and TRAF6 is a prerequisite for inducing the production of inflammatory cytokines (e.g., IL-6 and TNF) by innate immune cells, whereas the interaction between CD40 and TRAF2/3/5 is not.^25^ This effect of CD40-TRAF6 signaling is mediated by extracellular signal-regulated kinase (ERK) and NF-κB pro-inflammatory signaling.^25^ The SMI of CD40-TRAF6 signaling we used does not bind to the TRAF6 binding sites on IRAKs, leaving IL-1R/TLR signaling unaffected.^54^ Although multiple studies have demonstrated that CD40-TRAF6 signaling is involved in various immunological functions in macrophages and monocytes, the role of CD40-TRAF6 as a modifier of trained immunity had not yet been described.^55^ Our transcriptomic and epigenetic analyses indicate that TRAF6 signaling modulates trained immunity via NF-κB, which is known to regulate gene transcription through epigenetic modulation.^56^

By suppressing trained immunity, CD40-TRAF6 inhibition might serve as a potential treatment strategy for pathological conditions associated with trained immunity. To further explore this, we investigated the effect of CD40-TRAF6 blockade in a murine heart transplantation model. Previous studies indicated that ischemia-reperfusion injury, which inevitably occurs in organ transplantation, can result in the release of trained immunity-inducing DAMPs.^7,57,58^ Trained immune cells’ enhanced capacity for cytokine production may subsequently aggravate alloreactive T cell responses, contributing to graft rejection.^59^ IL-6 and TNF synergistically suppress the tolerizing effects of regulatory T (Treg) cells in allogeneic immune responses.^60^ In a previous study, we showed that suppressing trained immunity with NBs loaded with an mTOR inhibitor promotes immunologic graft acceptance.^11,61^ Our current study combined myeloid-directed CD40-TRAF6 inhibition with co-stimulatory inhibition using CTLA4-Ig. CTLA4-Ig is a therapy used clinically to prevent allograft rejection. Although favorable outcomes were reported in clinical trials, this approach suffers from a high risk of acute rejection compared to calcineurin-inhibitor-based treatments.^62,63^ A possible explanation is that CTLA4-Ig administration suppresses Treg expansion, which was observed in major histocompatibility complex class II-mismatched murine transplantation models.^64,65^ In humans, CTLA4-Ig therapy is also associated with reduced FOXP3 expression and decreased suppressive capacity of CD4^+^CD25^+^ T cells in kidney transplant patients.^66^ As inhibition of CD40-TRAF6 signaling reduced pro-inflammatory cytokine production in monocytes *in vitro*, we hypothesized that myeloid CD40-TRAF6 inhibition could have a synergistic effect with CTLA4-Ig therapy on graft acceptance. Indeed, we found that myeloid CD40-TRAF6 inhibition reinforces the previously observed positive effects of CTLA4-Ig on graft survival.^67^

In conclusion, our study reveals that trained immunity is potentiated through cellular interactions between T cells and monocytes, with CD40-TRAF6 signaling playing a pivotal role. Blocking CD40-TRAF6 helped identify modifying roles for CD40 signaling in trained immunity at transcriptional, metabolic, and epigenetic levels. The significance of CD40 signaling in trained immunity is corroborated by the association of SNPs near the *CD40* gene with trained immunity-induced cytokine responses. We further investigated the potential of myeloid CD40-TRAF6 inhibition in a heart transplantation mouse model and showed that inhibiting CD40-TRAF6 in myeloid cells effectively promotes graft acceptance when combined with CTLA4-Ig-mediated co-stimulatory blockade. With this, we show that CD40-TRAF6 blockade, which inhibits trained immunity *in vitro*, holds potential as a novel therapeutic approach for disorders characterized by aberrant innate immune activation, including organ transplantation.

### Limitations of the study

We investigated the role of T cells in trained immunity. In our experiments, we could not rule out that memory T cell responses from donor-derived PBMCs played a role, and we did not characterize the T cell subsets involved in mediating trained immunity induction. Regarding potential technical limitations, there is a chance that in our *in vitro* trained immunity assay, the removal of the training stimulus was not complete, although any remaining stimulus would likely be at very low concentrations. In our study, we applied myeloid CD40-TRAF6 inhibition in a murine heart transplant model and demonstrated that CD40-TRAF6 inhibition can be used to extend graft survival. However, the role of trained immunity in mediating transplant rejection was not demonstrated and should be further investigated in future studies.

## RESOURCE AVAILABILITY

### Lead contact

Further information and requests for resources and reagents should be directed to and will be fulfilled by the Lead Contact, Raphaël Duivenvoorden (Raphael.Duivenvoorden@radboudumc.nl).

### Materials availability

Materials are available upon reasonable request from the lead contact.

### Data and code availability

The RNA-seq and ChIP-seq datasets generated during this study are available at GEO Accession viewer (nih.gov) (GSE228184). This paper does not report original code. Any additional information required to reanalyze the data reported in this work paper is available from the lead contact upon request.

## STAR★METHODS

### EXPERIMENTAL MODEL AND STUDY PARTICIPANT DETAILS

#### Human subjects

For *in vitro* studies performed with human PBMCs (18–65 years old, male and female), buffy coats from healthy donors were obtained from Sanquin blood bank (Nijmegen) after written informed consent. No additional details are available on these subjects.

#### Human 300BCG cohort

The 300BCG cohort study was approved by the local ethics committee (CMO region Arnhem-Nijmegen, number NL58553.091.16). The 300BCG cohort consists of healthy Western European males (44%) and females (56%) between the age of 18–71 years. All participants gave written informed consent. Inclusion of volunteers and experiments were conducted following the principles expressed in the Declaration of Helsinki.

#### Mice

Female C57BL/6J (B6 WT, H-2b, JAX: 000664) and BALB/c (H-2d, JAX: 000651) mice were purchased from the Jackson Laboratory. Animals were maintained at the Center for Comparative Medicine and Surgery, Mount Sinai. Graft survival studies were performed with matched 8- to 11-week-old female mice (body weight, 18–21 g) in accordance with protocols approved by the Mount Sinai Animal Care and Utilization Committee. Animals were randomly assigned to experimental groups.

### METHOD DETAILS

#### PBMC isolation

PBMCs were isolated from buffy coats from healthy donors supplied by Sanquin blood bank, Nijmegen. PBMCs were isolated by Ficoll-Paque density gradient centrifugation (Lymphoprep, StemCell Technologies, Inc.). PBMCs were washed twice with PBS and were resuspended in RPMI 1640 culture medium supplemented with 1 mM pyruvate, 2 mM glutamax, and penicillin/streptomycin (all Thermo Fisher Scientific). Cells were counted using a Casy counter and analyzed with a Sysmex XN-450 automated hematology analyzer (Sysmex).

#### Monocyte and T cell isolation

Monocytes and T cells were isolated from PBMCs by negative MACS isolation using human Pan Monocyte Isolation Kit and human Pan T cell Isolation Kit (both Miltenyi Biotec) according to manufacturer’s instructions. Isolated monocytes and T cells were resuspended in RPMI 1640 culture medium supplemented with 1 mM pyruvate, 2 mM glutamax and penicillin/streptomycin and analyzed for purity with Sysmex XN-450 automated hematology analyzer. Purity of isolated cells was >90% and 95–99% for monocytes and T cells respectively.

#### Training experiments

Human adherent PBMCs and purified monocytes were trained as described previously.^19^ Briefly, PBMCs and monocytes were seeded into 96-well flat bottom plates in a density of 500.000 cells/well for PBMCs or 100.000 cells/well for monocytes, in RPMI 1640 culture medium supplemented with 1 mM pyruvate, 2 mM glutamax and penicillin/streptomycin. Cells were allowed to adhere for >1 h at 37°C, 5% CO_2_ (*v/v*). Cells were washed twice with PBS and where indicated, cells were treated with CD40L (MegaCD40L, 300 ng/mL, Enzo LifeSciences), CD40-TRAF6 signaling inhibitor (10 μM, Sigma), anti-PD-L1 (Ultra-LEAF Purified anti-human CD274, 40 μg/mL, Biolegend, RRID: AB_11149168) or CTLA4-Ig (40 μg/mL, BioXcell, RRID: AB_10949064) in culture medium supplemented with 10% FBS, for 1 h at 37°C, 5% CO_2_ (*v/v*). Subsequently, cells were stimulated with the primary stimuli HKCA (10^5^ cells/ml, InvivoGen), BCG vaccine (5 μg/mL, BCG-Bulgaria, Intervax), IFN-γ (20 ng/mL, InvivoGen) or culture medium for 24 h at 37°C, 5% CO_2_ (*v/v*). When CD40L, CD40-TRAF6 signaling inhibitor, anti-PD-L1 or CTLA4-Ig were present in culture prior to stimulation, these molecules remained in the culture medium during the stimulation period. After 24h, supernatant was collected, cells were washed three times with PBS and incubated in culture medium containing 10% FBS for 5 days at 37°C, 5% CO_2_ (*v/v*). Where indicated, IFN-γ stimulation was performed for 6 days instead of 24h. After 5 days, cells were restimulated with either RPMI as negative control, 1 μg/mL Pam3CSK4 (InvivoGen), or 10 ng/mL LPS (InvivoGen). After 24h, supernatant was collected for quantifying cytokine excretion.

#### Monocyte:T cell co-culture training experiments

Purified monocytes were seeded into 96-well or 24-well flat bottom plates at a density of 1*10^5^ or 5*10^5^ monocytes/well, respectively. Cells were allowed to adhere for 1 h at 37°C, 5% CO_2_ (*v/v*) and were washed twice with PBS. Purified autologous T cells were diluted in culture medium supplemented with 10% FBS and training stimuli (HKCA or RPMI) were added. T cell suspensions containing training stimuli were added to 24- or 96-wells to obtain indicated monocyte:T cell ratios. Where indicated, T cell suspensions were seeded into 96-well polyester transwell inserts that were placed in 24-wells plates. Monocyte:T cell ratio in co-cultures that were performed in transwell systems was 50:50. After stimulation, monocyte:T cell co-cultures were washed and cells were rested for 5 days. Restimulation of monocyte:T cell co-cultures was performed similarly as described for adherent PBMC and purified monocyte cultures.

#### Lactate dehydrogenase measurements

Lactate dehydrogenase (LDH) concentration was measured in supernatants of adherent PBMCs after 24h incubation with CTLA4-Ig, anti-PD-L1, CD40L or CD40-TRAF6 signaling inhibitor using CyQuant LDH Cytotoxicity Assay (Thermo Fisher Scientific). LDH concentration was calculated as a percentage of maximal LDH concentration in completely lysed cells according to the formula:

$$LDH\left(\%\;of\;\mathrm{maximum}\right)=\left(\frac{Compound\;induced\;LDH-Spontaneous\;LDH}{Maximal\;LDH-Spontaneous\;LDH}\right)\times 100$$

#### Cytokine quantification

Cytokine excretion was measured in supernatant harvested from adherent PBMC and monocyte cultures, using ELISA kits for human TNF-α, IL-6, IL-1β and IFN-γ (R&D systems) according to manufacturer’s instruction.

#### Flow cytometry in PBMCs

Human PBMCs were seeded in 100 mm petri dishes or 6-wells plates in a density of 5*10^6^ cells/mL for PBMCs or 1*10^6^ cells/mL for monocytes for 1 h, washed 2 times with PBS and were trained with indicated stimuli and inhibitors according to the previously described protocol for performance of training experiments. After the 5-day resting period, cells were incubated in 0.48 mM EDTA in PBS for 30 min at 37°C, 5% CO_2_ (*v/v*). Cells were scraped, counted and seeded in a 96-wells round bottom plate with a density of 2.5*10^5^ cells/well. Cells were stimulated with 10 ng/mL LPS or RPMI culture medium, in presence of GolgiPlug protein transport inhibitor (BD Biosciences) for 6 h. Cells were stained with Fixable Viability Stain 620 (BD Biosciences) according to manufacturer’s instructions. Cells were washed 2 times with PBS containing 1% bovine serum albumin (BSA, Sigma) and incubated in 10% heat-inactivated human serum (Serana) for blocking for 30 min on ice in dark. Antibody mixes for extracellular staining were added and incubated for 30 min on ice in dark. Antibodies used were anti-CD45-BV510 (Biolegend, 1:100, RRID: AB_2561383), anti-CD14-PE/Cyanine-7 (eBioscience, 1:100, RRID: AB_1582276), anti-CD16-FITC (eBioscience, 1:100, RRID: AB_10805747), anti-CD3-APCCy7 (Biolegend, 1:100, RRID: AB_2563410), anti-CD11b-BV785 (Biolegend, 1:100, RRID:AB_2563794), anti-CD40 BV785 (Biolegend, 1:100, RRID: AB_2566211), anti-CD86 BV650 (Biolegend. RRID: AB_11126752), anti-CD11c PE/Cyanine5.5 (Biolegend, 1:100, RRID: AB_493578) and anti-HLA-DR PE (Beckman Coulter, 1:100, RRID: AB_131284). When antibodies with a Brilliant Violet conjugate were included in the antibody mix, 10% Brilliant Stain Buffer (BD Biosciences) was present in the antibody mix. For intracellular cytokine staining, cells were washed 2 times with PBS containing 1% BSA and were resuspended, fixated and permeabilized using Fixation/Permeabilization Solution Kit (BD Biosciences) according to manufacturer’s instructions. Cells were incubated in antibody mixes containing anti-IL-6-APC (BD Biosciences, 1:25, RRID: AB_10679121) and anti-TNF-BV650 (Biolegend, 1:50, RRID: AB_2562741) for 30 min on ice in the dark. Cells were washed 2 times with PBS containing 1% BSA. Cells were resuspended in PBS 1% BSA and acquired with ACEA Novocyte 3000 (Agilent). Analyses were performed in FlowJo v10 Software (BD Biosciences).

#### Seahorse experiments

Human adherent PBMCs were treated with 10 μM CD40-TRAF6i (Sigma) or RPMI culture medium containing 10% FBS for 1 h at 37°C, 5% CO_2_ (*v/v*). Subsequently, cells were treated with HKCA (InvivoGen) in presence or absence of CD40-TRAF6i, or RPMI culture medium containing 10% FBS (negative control) for 24 h at 37°C, 5% CO_2_ (*v/v*). Stimuli and inhibitors were washed away and cells were rested for 5 days in culture medium containing 10% FBS. After resting, cells were washed with PBS and incubated in 0.48 mM EDTA (Sigma) in PBS for 30 min at 37°C, 5% CO_2_ (*v/v*). PBMCs were scraped, counted, and seeded in a density of 100.000 cells per well in quintuple, in XF96 microplates (Agilent Technologies). In an alternative approach to investigate metabolic activity specifically in monocytes, monocytes were isolated from the scraped PBMCs by means of negative MACS isolation using human Pan Monocyte Isolation Kit (Miltenyi Biotec). Monocytes were then counted and seeded in a density of 100.000 cells per well in quintuple in XF96 microplates. PBMCs or isolated monocytes were allowed to adhere for 1 h at 37°C, 5% CO_2_ (*v/v*). Culture medium was removed and cells were incubated for 45–60 min in a CO_2_-free incubator (37°C) in nonbuffered DMEM lacking glucose (Sigma Aldrich), supplemented with 0 mM or 11 mM D-Glucose (Sigma Aldrich), 0 mM or 1 mM pyruvate (Sigma Aldrich) and 1 mM or 2 mM L-Glutamine (Sigma Aldrich), dependent on the metabolic assay performed. Oxygen consumption and extracellular acidification were measured using a Seahorse XF96 Extracellular Flux Analyzer (Agilent Technologies) at 37°C. The Seahorse XF Cell Mito Stress Test was performed to analyze the cells’ oxygen consumption rate (OCR). During this assay, cells were treated with 1 μM oligomycin (Sigma Aldrich), 1 μM Carbonyl cyanide-4-(trifluoromethoxy)phenylhydrazone (FCCP, Sigma Aldrich) and a combination of 2.5 μM antimycin A (Sigma Aldrich) and 1.25 μM rotenone (Sigma Aldrich), at indicated time points. Dynamics in glycolytic activity were analyzed based on the extracellular acidification rate (ECAR) measured in the Seahorse XF Cell Glyco Stress Test. In this test, cells were treated with 11 mM glucose, 1 μM oligomycin and 22 mM 2-Deoxy-D-Glucose (Sigma Aldrich) at indicated time points.

#### Chromatin immunoprecipitation (ChIP)

Monocytes isolated from adherent after the 5-day resting period of training experiments, were resuspended in RPMI culture medium containing 1% formaldehyde (Sigma Aldrich) and incubated for 10 min at RT. Formaldehyde was quenched using 125 mM glycine, which was incubated for 5 min at RT. Monocytes were washed twice in cold PBS supplemented with a protease inhibitor cocktail (Roche). Cell pellets were snap frozen in liquid nitrogen and stored at −70°C until further use. Cells and nuclei were lysed using Magna ChIP Cell Lysis buffer and Nuclear Lysis buffer (Merck Millipore) according to manufacturer’s instructions. Lysates were sonicated using a Bioruptor pico sonicator (Diagenode; 10 cycles, 30 s on, 30 s off, at 4°C) in a density of 1.5*10^7^ cells/mL and spun down. Sheared chromatin was collected and fragment sizes were determined by DNA purification using MinElute Reaction Cleanup Kit (Qiagen) followed by gel electrophoresis. Immunoprecipitation was performed using the Magna ChIP A Chromatin Immunoprecipitation Kit (Merck Millipore) according to manufacturer’s instructions. In short, sonicated lysates derived from 5*10^5^ cells were incubated overnight with 1 μg H3K4me3 antibody or H3K27ac (Diagenode, RRID: AB_2616052 and RRID: AB_2637079) and protein A magnetic beads at 4°C on a rotating platform. Protein A beads-antibody/chromatin complexes were washed with buffers provided by the Magna ChIP kit for 5 min at 4°C on a rotating platform. Chromatin was eluted by incubation in Elution buffer supplemented with proteinase K (Magna ChIP kit, Merck Millipore) for 2 h at 62°C, shaking, followed by 10 min at 95°C. DNA was purified and eluted in 25 μL milliQ.

#### ChIP library preparation and sequencing

ChIP-seq libraries were prepared using the Kapa Hyper Prep Kit according to manufacturer’s instructions, with the following modifications. For adaptor ligation of each sample, 2.5 mL of the NEXTflex adaptor stock (600 nM, Bioo Scientific) was used. Libraries were amplified with 12–15 PCR cycles followed by a double post-amplification clean-up to ensure proper adapter removal. Samples were analyzed for purity using a High Sensitivity DNA Chip on a Bioanalyzer 2100 system (Agilent). Libraries were paired-end sequenced to a read length of 42 bp on an Illumina NextSeq500 (Illumina).

#### RNA isolation and RNA sequencing

RNA was isolated from 1*10^6^ monocytes using RNeasy Mini kit (Qiagen) including DNAse I digestion according to manufacturer’s instructions. RNA bulk sequencing was performed by Single Cell Discoveries (Utrecht, the Netherlands) with a sequencing depth of 20 million reads/sample. Library preparation was performed according to the CEL-seq2 protocol.^68^ Sequencing was performed on a Nextseq500 (Illumina).

#### CD40-TRAF6i-NB production and characterization

CD40-TRAF6i-loaded nanobiologics (CD40-TRAF6i-NBs) were formulated using a procedure based on our previous work.^11,24^ The CD40-TRAF6 inhibitor 68770028 (0.60 mg, 2.40 μmol, CAS no. 433249–94-6, MedChemExpress) was combined with 1-myristoyl-2-hydroxy-snglycero-phosphocholine (MHPC, 0.98 mg, 2.10 μmol, Avanti Polar Lipids) and 1,2-dimyristoyl-*sn*-glycero-3-phosphatidylcholine (DMPC 8.51 mg, 12.6 μmol, Avanti Polar Lipids) and dissolved in chloroform (5.0 mL), and dried under vacuum to yield a lipid film. A PBS solution of human apolipoprotein A1 (apoA1, 1 mg from a 3.70 mg/mL solution) was added. This apoA1 was purified from human HDL cholesterol concentrate (BioResource Technology). After incubating the suspension at 37°C for 2 h additional PBS was added until a total volume of 5.0 mL was reached. The solution was sonicated for 30 min using a Branson SFX 150 tip sonicator equipped with a 3/32″ microtip and operating at 65% output with pulse on for 3.5 s followed by pulse off for 2.0 s, while the sample was cooled in an ice bath. The opaque nanoparticle emulsion was concentrated to approximately 1.0 mL by centrifugal filtration using a 10 kDa MWCO Vivaspin tube. PBS (5.0 mL) was added and the mixture was again concentration to 1.0 mL, this was repeated twice. The resulting nanotherapeutics were sterilized using a 0.22 μm PES syringe filter, resulting in CD40-TRAF6i-NBs. Dynamic light scattering was performed in order to determine the hydrodynamic diameter and dispersity of the NBs. The indicated hydrodynamic diameter and dispersity represent the mean values for the multiple TRAF6i-NB batches that were used throughout experiments. Measurements per batch were performed in duplicate. Dynamic light scattering indicated a number-based mean size of 22 nm and a polydispersity of 0.26, in line with our previous results.^24^ The CD40-TRAF6i concentration in was determined in quadruplicate by UV-Vis spectrophotometry at λ = 378 nm. A standard curve was prepared using the concentration of non-encapsulated TRAF6i and the measured OD values measured for these concentrations. The amount of encapsulated drug was interpolated from this standard curve. Total encapsulated mass was quantified, taking into account the volume in which the compound was dissolved, and encapsulation efficiency was determined by comparing the encapsulated mass to the amount of compound that was used for the preparation of nanobiologics. Typical drug concentrations were 1.0 mg/mL and drug incorporation efficiencies were approximately 91%. To obtain fluorescently labeled CD40-TRAF6i-NBs (used for flow cytometry studies), 3,3′-dioactadecyloxacarbocyanine perchlorate [DiOC18(3); 0.25 mg] was dissolved in the chloroform mixture before drying the lipid film. The DiO concentration in the nanotherapeutics was determined by HPLC against a calibration curve of bare DiO.

#### Radiolabeling of CD40-TRAF6i-NBs

CD40-TRAF6i-loaded nanobiologics (CD40-TRAF6i-NBs) were radiolabeled with ^89^Zr according to previously described procedures.^69^ Briefly, to a solution of CD40-TRAF6i-NBs (1.06 mg of ApoA1, 1 eq, 0.6 mL), carbonate buffer was added to reach pH = 8.5. A solution of deferoxamine (DFO)-NCS (Macrocycles CAS no. 1222468–90-7), which a widely used chelator for labeling of zirconium-89 (^89^Zr), in DMSO (85 μg, 3 eq, 27 μL) was added in 5 μL portions.^70,71^ The reaction was incubated at 37°C for 1 h and manually shaken every 15 min. The solution was then washed 3 times with PBS using a vivaspin 10 kDa MWCO tubes. Then, radiolabeling with ^89^Zr was achieved by incubating the DFO-bearing nanoparticles with ^89^Zr-oxalate in PBS (pH = 7.1) at 37°C for 1 h. ^89^Zr-labeled-CD40-TRAF6i-NBs were purified by centrifugal filtration using 10 kDa MWCO tubes. The radiochemical yield was 84%.

#### Vascularized heterotopic heart transplantation

BALB/c hearts were transplanted as fully vascularized heterotopic grafts into C57BL/6J mice as previously described.^11,72^ Hearts were transplanted into recipients’ peritoneal cavities by establishing end-to-side anastomosis between donor and recipient aortae and end-to-side anastomosis between the donor pulmonary trunk and the recipient inferior vena cava. CD40-TRAF6i-NBs (5 mg/kg) or PBS were intravenously injected prior to surgery, as well as at days two and five post-transplantation, as performed in a previous graft survival experiment.^11^ CTLA4-Ig (0.13 mg/kg) was injected intraperitoneally prior to surgery, in line with a previous study.^67^ Cardiac allograft survival was assessed daily using the Visual Sonics Vevo 2100 Micro-Ultrasound imaging system. Rejection was defined as the complete cessation of cardiac contraction.

#### CD40-TRAF6i-NB pharmacokinetics and biodistribution

On day 5 after transplantation, untreated C57BL/6J mice heterotopically transplanted with BALB/c hearts were injected with ^89^ZrCD40-TRAF6i-NBs (177.3 ± 13.7 μCi) in 150–200 μL PBS via the tail vein. At predetermined time points (1, 5, 15, and 30 min, and 1, 2, 4, and 24h) blood samples (5–10 μL) were collected, weighed, and measured for radioactivity content using a Wizard^2^ 2480 automatic gamma counter (PerkinElmer). Data were converted to %ID/g, plotted in a time-activity curve, and fitted using a non-linear two-phase decay regression in Prism GraphPad (GraphPad Software Inc.). Finally, a weighted blood radioactivity t_1/2_ was calculated. At 24h, animals were anesthetized using 1.0% isoflurane in O_2_ at a flow rate of ~1.0 L/min. The PET/CT scans were performed using a Mediso nanoScan PET/CT (Mediso). A whole-body CT scan was acquired (energy, 50 kVp; current, 180 mAs; isotropic voxel size, 0.25 mm) followed by a 30 min PET scan. Reconstruction was performed with attenuation correction using the TeraTomo 3D reconstruction algorithm from the Mediso Nucline software. The coincidences were filtered with an energy window between 400 and 600 keV. The voxel size was isotropic with 0.4-mm width, and the reconstruction was applied for four full iterations, six subsets per iteration. Scans were analyzed in OsiriX MD 13.0.2.^73^ Immediately after PET-CT, the mice were sacrificed and perfused with PBS (20 mL). Tissues of interest were collected, weighed, and gamma-counted. Values were corrected for decay and expressed as a percentage of injected dose per gram of tissue (%ID/g).

#### Assessment of uptake of DiO-NBs by flow cytometry

Untreated C57BL/6J mice heterotopically transplanted with BALB/c hearts were injected with nanobiologics containing highly lipophilic DiO (DiO-NBs) on day 5 after transplantation, dosed at 0.5 mg of DiO per mouse kilogram, via tail vein injection. 24h later, animals were anesthetized with isoflurane and perfused with cold PBS (20 mL). Femurs, spleen, and graft were collected and stored in cold PBS. Bone marrow cells were flushed out of femurs and strained through a 70 μm strainer. Spleens were minced and meshed through a 70 μm strainer. Hearts were fragmented and incubated in 42.1 U/mL DNase, 60 U/mL Hyaluronidase, 125 U/mL Collagenase XI, 450 U/mL Collagenase I (all Sigma), 0.02 M HEPES buffer (Corning) in PBS for 1 h at 37°C, shaking at 50 rpm in the dark. Cells of grafts were then triturated and passed through a 70 μm strainer. Bone marrow, spleen, and graft single-cell suspensions were incubated with lysis buffer (BD Biosciences) for 1 min and washed with PBS. Single-cell suspension were stained for flow cytometry to determine DiO uptake in different leukocyte subsets. DiO-NBs were detected in the fluorescein isothiocyanate (FITC) channel.

#### Staining of blood leukocytes in allografts

To stain blood leukocytes in allografts of C57BL/6J mice treated with CD40-TRAF6i-NB and CTLA4-Ig 100 days post-transplantation, mice were intravenously injected with 1.5 μg anti-mouse CD45-BV711 (RRID: AB_2564383) in PBS 100 days after heterotopic heart transplantation. CD45-BV711 was allowed to circulate for 5 min. Mice were anesthetized and perfused with PBS, and native and transplanted hearts were collected. Single-cell suspension were prepared as described earlier.

#### Flow cytometry staining on murine tissues

Single-cell suspensions derived from grafts, native hearts, spleens and bone marrow were stained with LIVE/DEAD Fixable Aqua Dead Cell stain kit (Invitrogen) according to manufacturer’s instructions. After washing, cells were blocked for 10 min with anti-CD16/CD32 (BD Pharmingen, 1:220, RRID: AB_394657), followed by the incubation with the antibody cocktail for 30 min on ice and protected from light. Antibodies were diluted in FACS buffer (Dulbecco’s PBS complemented with 1% FBS, 1 mM EDTA, 0.5% bovine serum albumin, and 0.1% NaN3) with 10% Brilliant Stain Buffer (BD Biosciences). Antibodies used were anti-CD45-APC-eFluor780 (Thermo Fisher Scientific, 1:840, RRID: AB_1548781), anti-Ly-6C-BV570 (Biolegend, 1:840, RRID: AB_2562617), anti-Ly-6G-eFluor450 (Invitrogen, 1:420, RRID: AB_2637124), anti-CD11b-PerCP-Cy5.5 (BioLegend, 1:420, AB_893233), anti-CD19-PE (BD Biosciences, 1:420, RRID: AB_395050), anti-CD3-APC (BioLegend, 1:420, RRID: AB_2561455), and anti-CD115-BV421 (BioLegend, 1:420, RRID: AB_2562667), anti-CD19-BUV395 (BD Biosciences, 1:210, RRID: AB_2739418),. anti-CD3-BUV805 (BD Biosciences, 1:210, RRID: AB_2871285), anti-CD4-BV750 (Biolegend, 1:210, RRID: AB_2734150), anti-CD8a-PerCP (Biolegend, 1:210, RRID: AB_893423), anti-CD44-APC (Biolegend, 1:672, RRID: AB_312963), anti-CD11b-SparkYG 593 (Biolegend, 1:210, RRID: AB_2892261) and anti-F4/80-PE (eBioscience, 1:210, RRID: AB_465923). Data were acquired on a Cytek Aurora Flow Cytometer (5L configuration). Analyses were performed in FlowJo v10 Software (BD Biosciences).

### QUANTIFICATION AND STATISTICAL ANALYSIS

#### In vitro experiment data analysis

Statistical analyses were performed in GraphPad Prism 9.1.2. Data are presented as mean ± SEM if not specified otherwise. Sample sizes per experiment are indicated as ‘n’ in the figure legends. Samples were compared using paired t-tests or paired two-Way ANOVA with Šidák’s post-test if not specified otherwise. We did not perform normality tests because of the low power of normality tests for assessing the normal distribution for small sample sizes. Trained immunity experiments in larger sample sizes show a normal distribution. According to the central limit theory, which states that the averages of random samples from a random distribution will themselves have a normal distribution, we assume that our samples are normally distributed.^74^

#### RNA sequencing data analysis

Differential gene expression analysis was performed on counts of 3 samples per treatment group using the DESeq2 package in Rstudio.^75,76^ Samples were paired for donor. Differentially Expressed Genes (DEGs) were defined as fold change (FC) < 0.5 or FC > 2 and FDR <0.1.^77^ Enrichment of Gene Ontology Biological Processes among DEGs was analyzed using String-DB.^78^

#### Gene set enrichment analysis

Gene set enrichment analyses (GSEA) were performed on normalized counts of 3 samples per treatment group using GSEA software v4.1.0 provided by Broad Institute.^79^ GSEA was performed using the gene sets from HALLMARK database of the Molecular Signature database (MSigDB).^80,81^ Analyses were conducted with 1000 gene set permutations and with the following settings: Metric for ranking genes: Signal2Noise; Remap/Collapse to gene symbols: Collapse; Enrichment statistic: weighted; Normalization mode: meandiv. For each gene set, a Normalized Enrichment Score (NES) was calculated. Gene sets for which FDR <0.1 were considered to be enriched.

#### Gene regulatory network inference

The RNA-sequencing data above was processed. The gene-count matrix was subjected to edgeR and variably expressed genes were identified by ANOVA-like analysis. The gene-count matrix was normalized by counts per million and kept only variably expressed genes. Gene regulatory network inference was conducted by GENIE3.^82^ Potential DNA binding factors were subjected to GENIE3 as regulators. The resulting network was visualized in Cytoscape.^83^

#### Seahorse metabolic parameters analysis

Metabolic parameters were calculated from the OCR and ECAR measured with Seahorse technology as follows. The basal respiration was calculated as the mean OCR before adding oligomycin. ATP-linked respiration was calculated as the mean OCR of timepoints 1–4 subtracted by the mean OCR of timepoints 5–7. The maximal respiration was calculated as the mean OCR of timepoints 8–10 subtracted by the average OCR of timepoints 11–13. The spare respiratory capacity was calculated as the mean OCR of timepoints 8–10 subtracted by the mean OCR of timepoints 1–4. Non-mitochondrial oxygen consumption was calculated as the mean OCR of timepoints 11–13. Glycolysis was calculated as the mean ECAR of timepoints 5–7 subtracted by the mean ECAR of timepoints 1–4. The glycolytic capacity was calculated as the mean ECAR of timepoints 8–10 subtracted by the mean ECAR of timepoints 1–4. Non-glycolytic acidification was calculated as the mean ECAR of timepoints 1–4.

#### ChIP-sequencing data analysis

ChIP-sequencing data were aligned to human genome hg38 with BWA.^84^ Samtools was used to filter reads with a quality score lower than 20. PCR duplicates were removed with Picard.^85^ Peaks were identified with MACS 2.2.6 in paired-end mode and ‘call-summits’ enabled at a false discovery rate of 0.01.^86^ A union of all identified peaks was generated with BEDTools, which was used to count reads per peak in all samples.^87^ DESeq2 package in R was used to identify significant changes in detected peaks among treatments.^75^ Samples derived from one donor were analyzed pairwise. Significant dynamics in peaks was defined by FC < 0.5 or FC > 2 and an FDR >0.10. Genomic Regions Enrichment of Annotations Tool (GREAT) was used for assigning ChIP peaks to closely located genes, and for identifying significantly enriched Gene Ontologies.^88^ Bigwig files were created using Deeptools.^89^ H3K4me3 and H3K27ac marks were visualized using UCSC Genome Browser.^90^

#### Training in the 300BCG cohort and QTL mapping

*Ex vivo* BCG and β-glucan training was performed using adherent PBMCs from 267 healthy individuals of Western European ancestry from the 300BCG cohort (NL58553.091.16) for whom we had genotype data. Adherent PBMCs were trained with RPMI (control), BCG vaccine or β-glucan (β−1,3-(D)-glucan, provided by Professor David Williams (College of Medicine, Johnson City, USA)) according to the protocol used for training experiments. TNF and IL-6 was measured 24h after LPS restimulation (O55:B5, Sigma Aldrich) as described in previous section. Fold changes were calculated compared to cytokine production upon LPS restimulation of RPMI-treated PBMCs.

*In vivo* training was performed by means of BCG vaccination of 278 participants of the 300BCG cohort. Participants were vaccinated intradermally in the left upper arm with a standard dose of 0.1 mL BCG (BCG-Bulgaria, InterVax). EDTA blood was collected before, 2 weeks and 3 months after BCG vaccination and PBMCs were isolated. Adherent PBMCs were stimulated with 5 × 10^6^ CFU/mL heat-killed *Staphylococcus aureus (S. aureus)* for 24h. TNF, IL-1β and IL-6 (R&D Systems) was measured in supernatant collected 24h, and IFN-γ (Luminex, Thermo Fisher) was quantified in supernatant collected 7 days after *S. aureus* stimulation according to manufacturer’s instructions. Fold changes were calculated compared to cytokine production by PBMCs that were collected from the same individual prior to BCG vaccination.

DNA samples of subjects were genotyped using the commercially available SNP chip, Infinium Global Screening Array MD v1.0 (Illumina). Genotype information on approximately 4 million single-nucleotide polymorphisms (SNPs) was obtained upon imputation (MAF >5% and R^2^ > 0.3 for imputation quality). Genetic outliers (*n* = 17) were removed before QTL mapping. First, raw cytokine levels were log-transformed and the ratio between trained and non-trained cytokine levels taken as the change of cytokine levels. The cytokine changes were mapped to genotype data using a linear regression model with age and sex as covariates. R-package Matrix-eQTL was used for cytokine QTL mapping.^91^ A linear regression model with age and sex as covariates was used to analyze the effect of genotype on the fold change of cytokine production in FTI-QLT analysis.

#### Graft survival analysis

For comparing graft survival in heterotopic heart transplantation experiments, Kaplan-Meier curves were plotted for allograft survival analysis, and differences between the groups were evaluated using a log rank test.

## Supplementary Material

1

## Figures and Tables

**Table:** KEY RESOURCES TABLE

REAGENT or RESOURCE	SOURCE	IDENTIFIER

Antibodies		

Anti-human CD16 FITC	eBioscience	Cat#11-0168-42; RRID: AB_10805747
Anti-human CD3 APC-Cy7	Biolegend	Cat#317342; RRID: AB_2563410
Anti-human CD11b BV785	Biolegend	Cat#301346; RRID:AB_2563794
Anti-human CD45 BV510	Biolegend	Cat#304035; RRID: AB_2561383
Anti-human CD14 PE/Cyanine7	eBioscience	Cat#25-0149-42; RRID: AB_1582276
Anti-human TNFα BV650	Biolegend	Cat#502938; RRID: AB_2562741
Anti-human IL-6 APC	BD Biosciences	Cat#561441; RRID: AB_10679121
Anti-human FoxP3 Pe-Cy7	Thermo Fisher Scientific	Cat#25-4776-42; RRID: AB_10804638
Anti-human CD4 Super Bright	Thermo Fisher Scientific	Cat#78-0047-42; RRID: AB_2744896
Anti-human CD8 PerCP	Biolegend	Cat#344708; RRID: AB_1967149
Anti-human CD40 BV785	Biolegend	Cat#334340; RRID: AB_2566211
Anti-human CD86 BV650	Biolegend	Cat#305428; RRID: AB_11126752
Anti-human CD3 BV605	Biolegend	Cat#300460; RRID: AB_2564380
Anti-human HLA-DR PE	Beckman Coulter	Cat#IM1639; RRID: AB_131284
Anti-human CD11c PE/Cyanine5.5	Biolegend	Cat#301610; RRID: AB_493578
Anti-mouse CD19 BUV395	BD Biosciences	Cat#565965; RRID: AB_2739418
Anti-mouse CD19 PE	BD Biosciences	Cat#553786; RRID: AB_395050
Anti-mouse CD3 BUV805	BD Biosciences	Cat#741982; RRID: AB_2871285
Anti-mouse CD3 APC	Biolegend	Cat#100236; RRID: AB_2561455
Anti-mouse CD4 BV750	Biolegend	Cat#100467; RRID: AB_2734150
Anti-mouse CD8a PerCP	Biolegend	Cat#100732; RRID: AB_893423
Anti-mouse/human CD44 APC	Biolegend	Cat#103012; RRID: AB_312963
Anti-mouse CD45 APC-eFluor 780	Thermo Fisher Scientific	Cat#47-0451-82; RRID: AB_1548781
Anti-mouse/human CD11b Spark YG 593	Biolegend	Cat#101281; RRID: AB_2892261
Anti-mouse/human CD11b PerCP-Cy5.5	Biolegend	Cat#101228; RRID: AB_893233
Anti-mouse Ly-6C BV570	Biolegend	Cat#128030; RRID: AB_2562617
Anti-mouse Ly-6G eFluor 450	Thermo Fisher Scientific	Cat#48-9668-82; RRID: AB_2637124
Anti-mouse F4/80 PE	Thermo Fisher Scientific	Cat#12-4801-82; RRID: AB_465923
Anti-mouse CD115 BV421	Biolegend	Cat#135513; RRID: AB_2562667
Anti-mouse CD45 BV711	Biolegend	Cat#103147; RRID: AB_2564383
Anti-mouse CD16/CD32 (Fc-block)	BD Pharmingen	Cat#553142; RRID: AB_394657
Rabbit polyclonal anti H3K4me3	Diagenode	Cat#pab-003-050; RRID: AB_2616052
Rabbit polyclonal anti-H3K27Ac	Diagenode	Cat#pab-196-050; RRID: AB_2637079
Ultra-LEAF^™^ Purified anti-human CD274 (B7-H1, PD-L1)	Biolegend	Cat#329716; RRID: AB_11149168
InVivoMAb Recombinant CTLA4-Ig (for *in vitro* studies)	BioXcell	Cat#BE0099; RRID: AB_10949064

Biological samples		

Human PBMCs from buffy coats	Sanquin Bloodbank	

Chemicals, peptides, and recombinant proteins		

Ficoll-Paque (Lymphoprep)	StemCell Technologies, Inc.	Cat#07861
Glutamax	Thermo Fisher Scientific	Cat#35050
Pyruvate	Thermo Fisher Scientific	Cat#11360
Penicillin/Streptomycin	Thermo Fisher Scientific	Cat#15140
RPMI 1640	Thermo Fisher Scientific	Cat#22409
Fetal Bovine Serum	Serana Europe GmbH	Cat#S-FBS-EU-025
EDTA	Sigma	Cat#E5134
Heat-killed Candida albicans	Invivogen	Cat#tlrl-hkca
Bacille Calmette-Guérin Vaccine	Intervax	Bulgaria strain
Lipopolysaccharide (*E.* coli, O55:B5), used in the 300BCG study	Sigma-Aldrich	Cat#L2880
Lipopolysaccharide Ultrapure (E.coli, O111:B4) used for *in vitro* studies	Invivogen	Cat#tlrl-3pelps
Pam3CSK4	Invivogen	Cat#tlrl-pms
Recombinant human IFN-gamma	Invivogen	Cat#rcyec-hifng
Bovine Serum Albumin	Sigma	Cat#A3803
CD40-TRAF6 signaling inhibitor (for *in vitro* studies)	Sigma	Cat#SML1160
Human MegaCD40L Protein	Enzo Lifesciences	Cat#ALX-522-110-C010
Brilliant Stain Buffer	BD Biosciences	Cat#563794
16% Formaldehyde	Sigma Aldrich	Cat#28908
DNase I	Qiagen	Cat#79254
cOmplete^™^, Mini, EDTA-free Protease Inhibitor Cocktail	Roche	Cat#11836170001
Heat-inactivated human serum	Serana	Cat#S-HU-EU-011
Oligomycin	Sigma Aldrich	Cat#O4876
Rotenone	Sigma Aldrich	Cat#R8875
Antimycin A	Sigma Aldrich	Cat#A8674
D-Glucose	Sigma Aldrich	Cat#G8644
DMEM	Sigma Aldrich	Cat#D5030
L-Glutamine	Sigma Aldrich	Cat#G3126
Sodium Pyruvate	Sigma Aldrich	Cat#P2256
Carbonyl cyanide-4-(trifluoromethoxy) phenylhydrazone (FCCP)	Sigma Aldrich	Cat#C2920
2-deoxy-D-glucose	Sigma Aldrich	Cat#D6134
LIVE/DEAD Fixable Aqua Dead Cell Stain Kit	Invitrogen	Cat#L34966
Fixable Viability Stain 620	BD Biosciences	Cat#564996
CD40-TRAF6 signaling inhibitor (for *in vivo* studies)	MedChemExpress	Cat#HY-110247
CTLA4-Ig (for *in vivo* studies)	MedChemExpress	Cat#HY-108829
DMPC	Avanti Polar Lipids	Cat#850345
MHPC	Avanti Polar Lipids	Cat#855575
DiO: DiOC_18_^3^	Invitrogen	Cat#D275
Human HDL cholesterol concentrate	BioResource Technology	Cat#H3025
Deoxyribonuclease I bovine	Sigma Aldrich	Cat#D5319
Hyaluronidase	Sigma Aldrich	Cat#H3506
Collagenase XI from *Clostridium histolyticum*	Sigma Aldrich	Cat#C7657
Collagenase I from *Clostridium histolyticum*	Sigma Aldrich	Cat#C0130
HEPES buffer	Corning	Cat#25-060-Cl
BD Pharm Lysing Buffer	BD Biosciences	Cat#555899

Critical commercial assays		

Human TNF-α DuoSet ELISA	R&D systems	Cat#DY210
Human IL-6 DuoSet ELISA	R&D systems	Cat#DY206
Human IFN-gamma DuoSet ELISA	R&D systems	Cat#DY285B
Human Pan T cell Isolation Kit	Miltenyi Biotec	Cat#130-096-535
Human Pan Monocyte Isolation Kit	Miltenyi Biotec	Cat#130-096-537
CyQUANT LDH Cytotoxicity Assay	Thermo Fisher Scientific	Cat#C20302
VersaComp Antibody Capture Beads Kit	Beckman Coulter	Cat#B22804
AccuCount Fluorescent Particles	Spherotech	Cat#ACFP-50-5
RNeasy Mini kit	Qiaqen	Cat#74106
Magna ChIP^™^ A Chromatin Immunoprecipitation Kit	Merck Millipore	Cat#17-408
MinElute Reaction Cleanup Kit	Qiaqen	Cat#28204
Fixation/Permeabilization Solution Kit with BD GolgiPlug^™^	BD Biosciences	Cat#555028
FoxP3 transcription factor staining set	eBioscience	Cat#00-5523-00

Deposited data		

*In vitro* trained monocyte ChIP-seq and RNA-seq data	This paper	GSE228184

Experimental models: Orqanisms/strains		

BALB/c mice	Jackson Laboratories	RRID: IMSR_JAX:000651
C57BL/6J mice	Jackson Laboratories	RRID: IMSR_JAX:000664
300BCG cohort (Human Functional Genomics Project)	N/A	https://www.humanfunctionalqenomics.orq

Software and alqorithms		

GraphPad Prism 9.1.2	GraphPad Software	N/A
R	R Team^76^	
FlowJo^™^ v10	BD Biosciences	N/A
NovoExpress software 1.5.6	Agilent	N/A
GSEA	Subramanian et al.^79^ Liberzon et al.^80^	
Samtools	Li et al.^84^	http://samtools.sourceforqe.net
BEDtools	Quinlan et al.^87^	http://code.qooqle.com/p/bedtools
DESeq2	Love et al.^75^	http://www.bioconductor.orq/packaqes/release/bioc/html/DESeq2.html
GREAT	McLean et al.^88^	http://qreat.stanford.edu/public/html/
BioRender		BioRender
OsiriXMD 13.0.2	Rosset et al.^73^	https://www.osirix-viewer.com/osirix/osirix-md/
GENIE3	Huynh-Thu et al.^82^	
Cytoscape	Franz et al.^83^	https://cytoscape.orq/
Deeptools	Ramirez et al.^89^	https://deeptools.readthedocs.io/en/develop/index.html
UCSC Genome Browser	Nassar^90^	https://qenome.ucsc.edu/index.html
STRING database	Szklarczyk^77,78^	https://strinq-db.orq/

## Highlights

- T cells modulate trained immunity induction in monocytes via CD40-TRAF6 signaling
- Blocking CD40-TRAF6 signaling inhibits trained immunity in monocytes *in vitro*
- SNPs in the proximity of *CD40* associate with trained immunity responses *in vivo*
- Myeloid-specific CD40-TRAF6 inhibition prolongs allograft survival *in vivo*
