# Supplementary material for: Trained immunity is regulated by T cell-induced CD40-TRAF6 signaling
Source: Cell Rep. Author manuscript; Available in PMC 2024 Nov 5. (PMC11536040; doi:10.1016/j.celrep.2024.114664)
Supplement: 1 [file NIHMS2025323-supplement-1.pdf]

Cell Reports, Volume *43*

Supplemental information

Trained immunity is regulated by T cell-induced CD40-TRAF6 signaling

Maaike M.E. Jacobs, Rianne J.F. Maas, Inge Jonkman, Yutaka Negishi, Willem Tielemans Zamora, Cansu Yanginlar, Julia van Heck, Vasiliki Matzaraki, Joost H.A. Martens, Marijke Baltissen, Michiel Vermeulen, Judit Morla-Folch, Anna Ranzenigo, William Wang, Martin Umali, Jordi Ochando, Johan van der Vlag, Luuk B. Hilbrands, Leo A.B. Joosten, Mihai G. Netea, Willem J.M. Mulder, Mandy M.T. van Leent, Musa M. Mhlanga, Abraham J.P. Teunissen, Nils Rother, and Raphaël Duivenvoorden

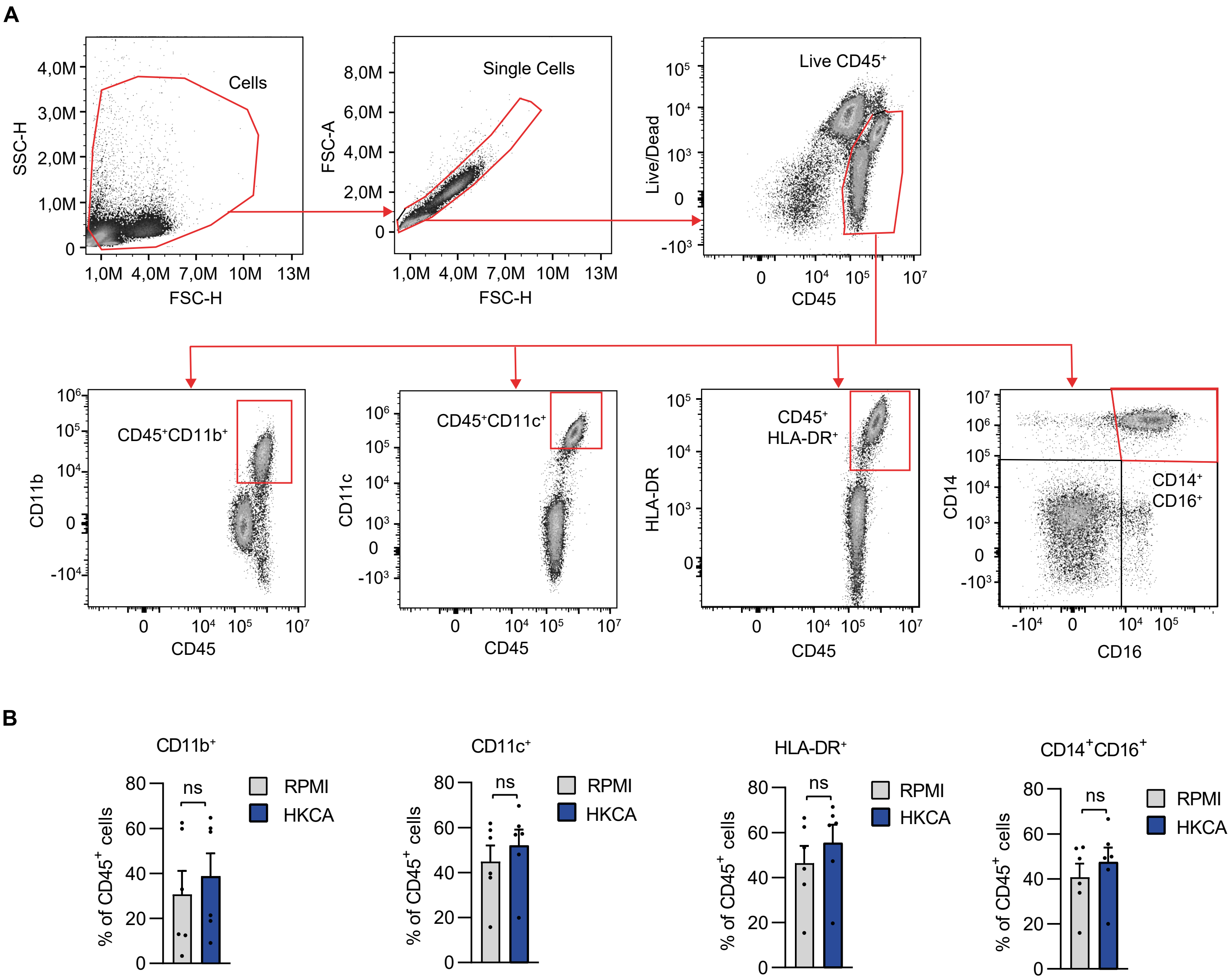
A
Cells
SSC-H
FSC-H
Single Cells
FSC-A
FSC-H
Live CD45+
Live/Dead
CD45
CD45+CD11b+
CD11b
CD45
CD45+CD11c+
CD11c
CD45
CD45+
HLA-DR+
HLA-DR
CD45
CD14+
CD16+
CD14
CD16
B
CD11b+
CD11c+
HLA-DR+
CD14+CD16+
% of CD45+ cells
ns
RPMI
HKCA

Supplementary Figure 1: Effect of HKCA-induced trained immunity on expression of myeloid differentiation markers. Related to Figure 1.

A: Gating strategy applied for determining expression of CD11b, CD11c, HLA-DR and CD14/CD16 in PBMCs 6 days after stimulation with RPMI or heat-killed *Candida albicans* (HKCA).

B: Expression of CD11b, CD11c, HLA-DR and CD14/CD16 in PBMCs 6 days after stimulation with RPMI or HKCA. Mean ± SEM are shown.

A

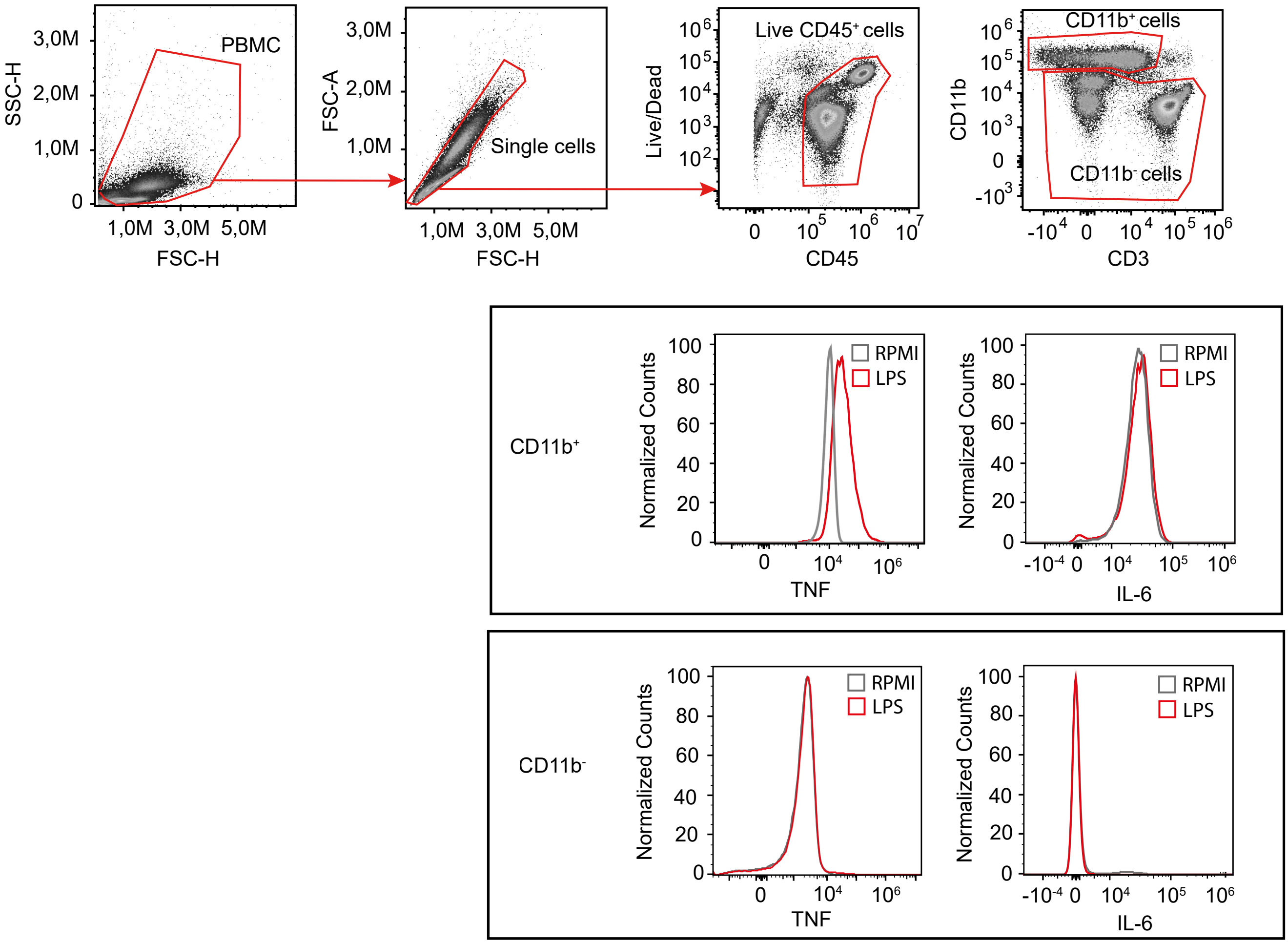

PBMC
SSC-H
FSC-H
Single cells
FSC-A
Live CD45+ cells
Live/Dead
CD45
CD11b+ cells
CD11b- cells
CD11b
CD3
CD11b+
CD11b-
Normalized Counts
TNF
IL-6
RPMI
LPS

B

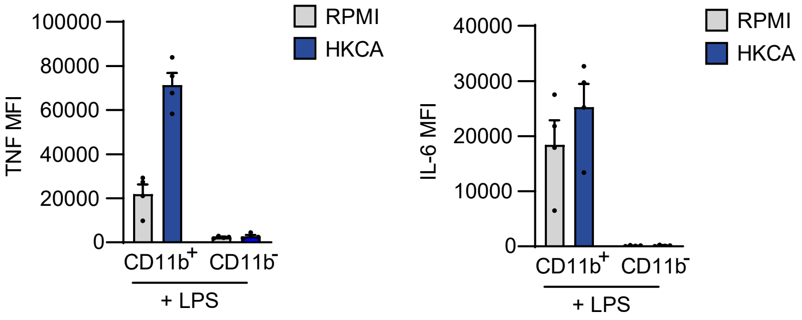

TNF MFI
IL-6 MFI
RPMI
HKCA
CD11b+
CD11b-
+ LPS

Supplementary Figure 2: TNF and IL-6 are produced by $CD11b^+$ cells of PBMCs. Related to Figure 1.

A: Gating strategy applied for determining TNF and IL-6 production in myeloid ($CD11b^+$) and non-myeloid ($CD11b^-$) cells in adherent PBMCs that were trained with RPMI and restimulated with RPMI medium or lipopolysaccharide (LPS) 5 days later.

B: Expression of TNF and IL-6 in untreated and heat-killed *Candida albicans* (HKCA)-treated $CD11b^+$ and $CD11b^-$ subsets of adherent PBMCs after restimulation with LPS 5 days after HKCA- or RPMI stimulation, determined with flow cytometry (n=3 donors). Mean ± SEM are shown. Data is shown as mean fluorescent intensity (MFI).

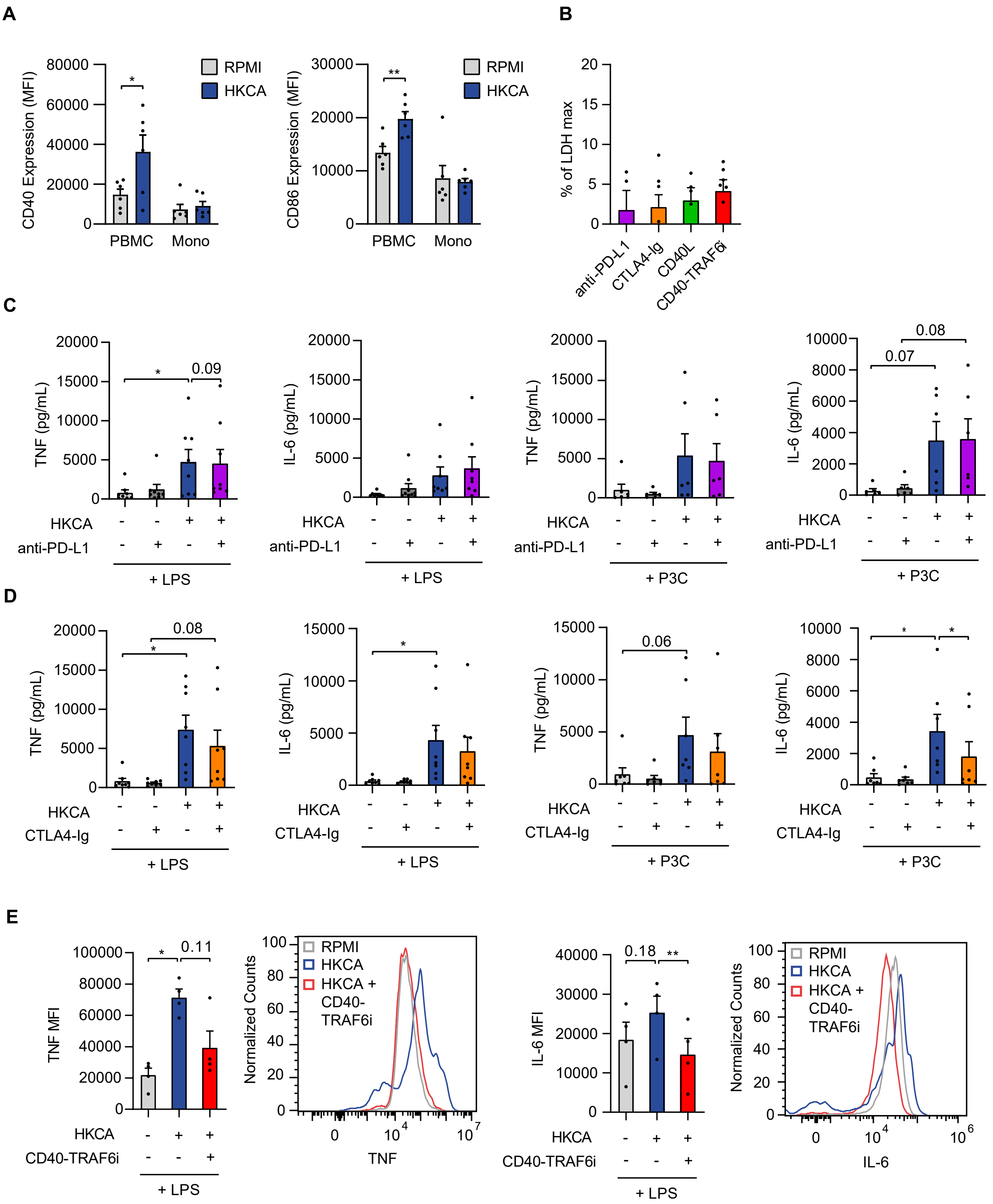
A
CD40 Expression (MFI)
80000
60000
40000
20000
0
PBMC
Mono
RPMI
HKCA
CD86 Expression (MFI)
30000
20000
10000
0
B
% of LDH max
anti-PD-L1
CTLA4-Ig
CD40L
CD40-TRAF6i
C
TNF (pg/mL)
IL-6 (pg/mL)
0.09
0.07
0.08
HKCA
anti-PD-L1
+ LPS
+ P3C
D
CTLA4-Ig
0.06
E
TNF MFI
100000
0.11
Normalized Counts
HKCA +
CD40-
TRAF6i
TNF
IL-6 MFI
0.18
IL-6
CD40-TRAF6i

Supplementary Figure 3: Effect of anti-PD-L1, CTLA4-Ig and CD40-TRAF6 signaling inhibitor on induction of trained immunity in PBMCs. Related to Figure 2.

A: Expression of CD40 and CD86 in monocytes present in adherent PBMCs, and purified monocytes that were not stimulated or stimulated with heat-killed *Candida albicans* (HKCA) for 24 hours, determined with flow cytometry (n=6 donors). Data is shown as mean fluorescent intensity (MFI).

B: Percentage of Lactate Dehydrogenase (LDH) in the supernatant of adherent PBMCs treated for 24 hours with CD40L (300 ng/mL), CD40-TRAF6i (10 µM), CTLA4-Ig (40 µg/mL) or anti-PD-L1 (40 µg/mL), compared to maximal LDH (n=4-7 donors).

C: TNF and IL-6 production in adherent PBMCs that were untreated, or treated with HKCA in the presence or absence of anti-PD-L1, upon restimulation with lipopolysaccharide (LPS) (n=8 donors) or Pam3CSK4 (P3C) (n=6 donors).

D: TNF and IL-6 production in adherent PBMCs that were untreated, or treated with HKCA in the presence or absence of CTLA4-Ig, upon restimulation with LPS (n=8 donors) or P3C (n=7 donors).

E: Expression of TNF and IL-6 in untreated and HKCA-treated adherent PBMCs in the presence or absence of CD40-TRAF6i 6 hours after restimulation with LPS, determined with flow cytometry (n=4). Data is shown as mean fluorescent intensity (MFI).

Mean ± SEM are shown. * $p < 0.05$, ** $p < 0.01$. Paired t tests (A), paired Two-Way ANOVA with Šidák's post-test (C-D) or paired One-Way ANOVA with Dunnett's correction (E) was used.

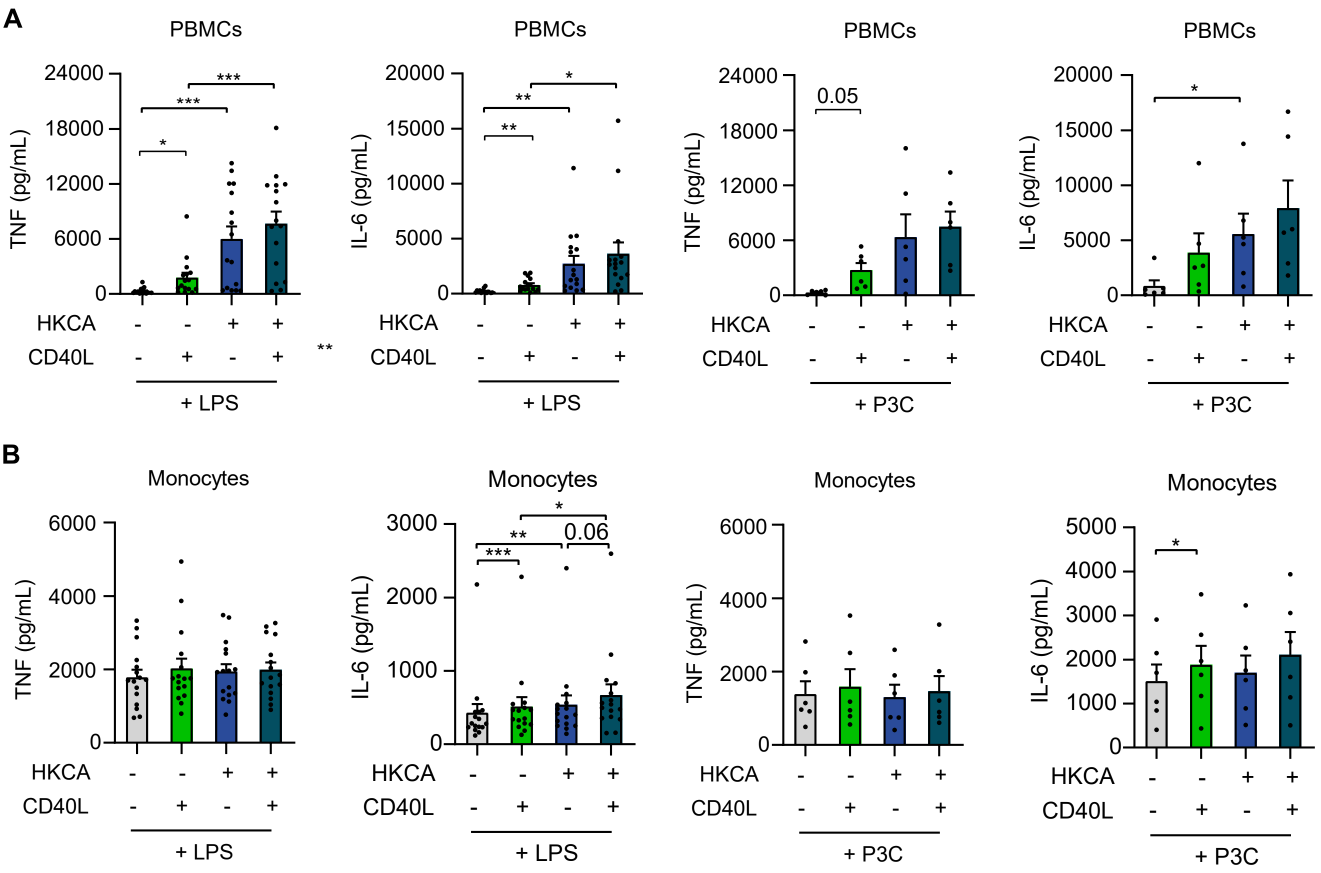
A
PBMCs
TNF (pg/mL)
24000
18000
12000
6000
0
*
***
***
HKCA - - + +
CD40L - + - +
+ LPS
**
PBMCs
IL-6 (pg/mL)
20000
15000
10000
5000
0
**
**
*
HKCA - - + +
CD40L - + - +
+ LPS
PBMCs
TNF (pg/mL)
24000
18000
12000
6000
0
0.05
HKCA - - + +
CD40L - + - +
+ P3C
PBMCs
IL-6 (pg/mL)
20000
15000
10000
5000
0
*
HKCA - - + +
CD40L - + - +
+ P3C
B
Monocytes
TNF (pg/mL)
6000
4000
2000
0
HKCA - - + +
CD40L - + - +
+ LPS
Monocytes
IL-6 (pg/mL)
3000
2000
1000
0
*
**
0.06
***
HKCA - - + +
CD40L - + - +
+ LPS
Monocytes
TNF (pg/mL)
6000
4000
2000
0
HKCA - - + +
CD40L - + - +
+ P3C
Monocytes
IL-6 (pg/mL)
5000
4000
3000
2000
1000
0
*
HKCA - - + +
CD40L - + - +
+ P3C

Supplementary Figure 4: Effect of CD40L stimulation on HKCA-induced training. Related to Figure 2.

A: TNF and IL-6 production in adherent PBMCs that were not treated or treated with heat-killed *Candida albicans* (HKCA) in presence or absence of CD40 ligand (CD40L) upon restimulation with lipopolysaccharide (LPS) (n=16 donors) or Pam3CSK4 (P3C) (n=6 donors).

B: TNF and IL-6 production in monocytes that were not treated or treated with HKCA in presence or absence of CD40L upon restimulation with LPS (n=16 donors) or P3C (n=6 donors).

Mean ± SEM are shown. * $p < 0.05$, ** $p < 0.01$, *** $p < 0.001$, paired Two-way ANOVA with Šidák's post-test.

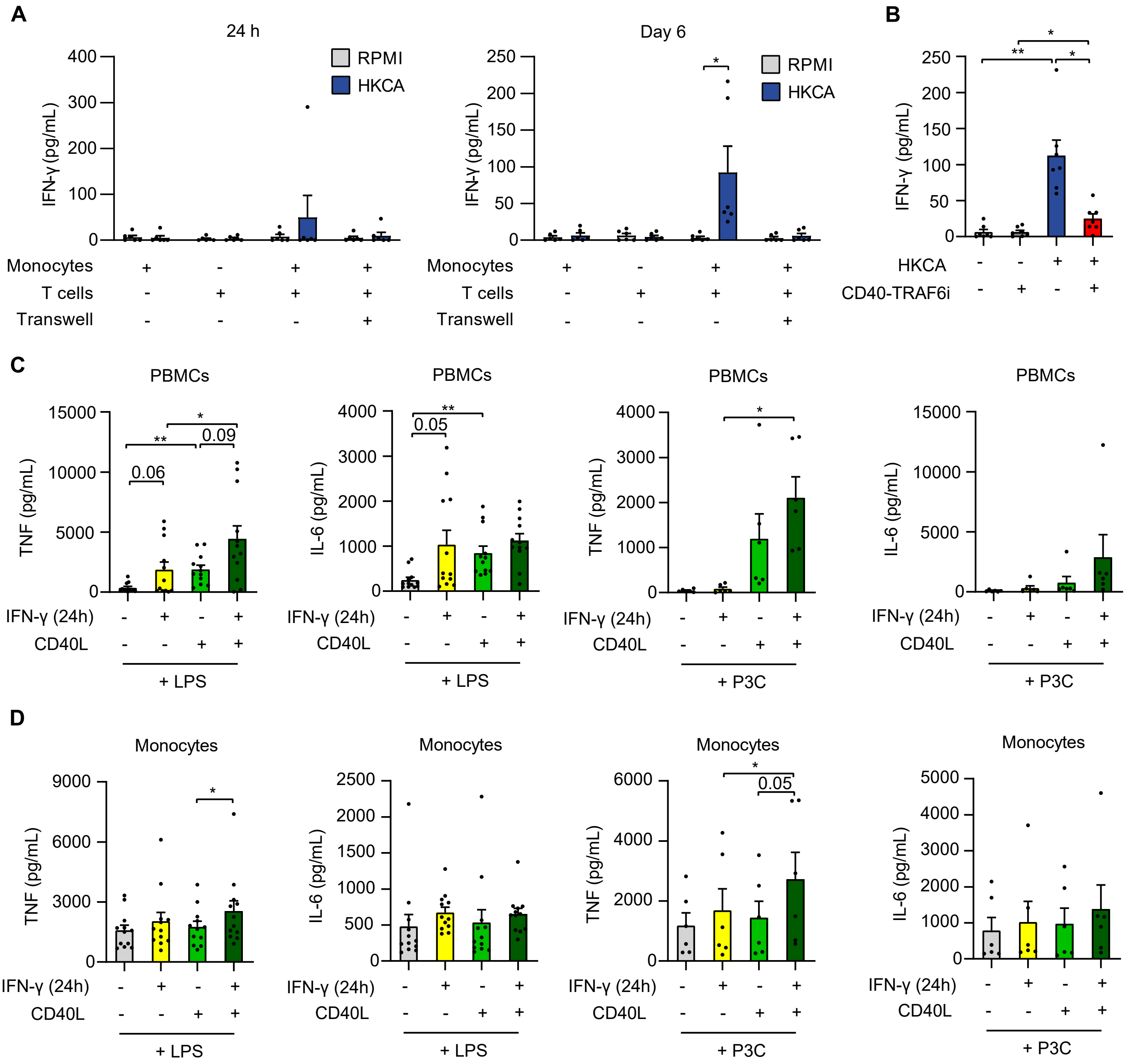
A
24 h
Day 6
RPMI
HKCA
IFN-γ (pg/mL)
Monocytes
T cells
Transwell
B
HKCA
CD40-TRAF6i
C
PBMCs
TNF (pg/mL)
IL-6 (pg/mL)
IFN-γ (24h)
CD40L
+ LPS
+ P3C
0.06
0.09
0.05
D
Monocytes

Supplementary Figure 5: IFN-γ stimulation for 24 hours does not induce training responses in PBMCs and monocytes. Related to Figure 2.

A: IFN-γ concentrations measured in the supernatant of monocytes, T cells or autologous monocyte:T cell co-cultures performed in presence or absence of a transwell, that were not treated or treated with heat-killed *Candida albicans* (HKCA), collected 24 hours or 6 days after stimulation with HKCA or RPMI (n=6 donors).

B: IFN-γ concentrations in supernatants of adherent PBMCs that were either not treated, HKCA-treated in the presence or absence of CD40-TRAF6i, measured in supernatant collected 6 days after stimulation (n=6 donors).

C: TNF and IL-6 production in adherent PBMCs treated for 24 h with IFN-γ in the presence or absence of CD40 ligand (CD40L), or RPMI, after restimulation with lipopolysaccharide (LPS) (n=12 donors) or Pam3CSK4 (P3C) (n=6 donors).

D: TNF and IL-6 production in monocytes treated for 24 h with IFN-γ in the presence or absence of CD40L, or RPMI, after restimulation with LPS (n=12 donors) or P3C (n=6 donors). Mean ± SEM are shown. * $p < 0.05$, ** $p < 0.01$. Paired t tests (A) and paired Two-way ANOVA with Šidák’s post-test (B-D) were used.

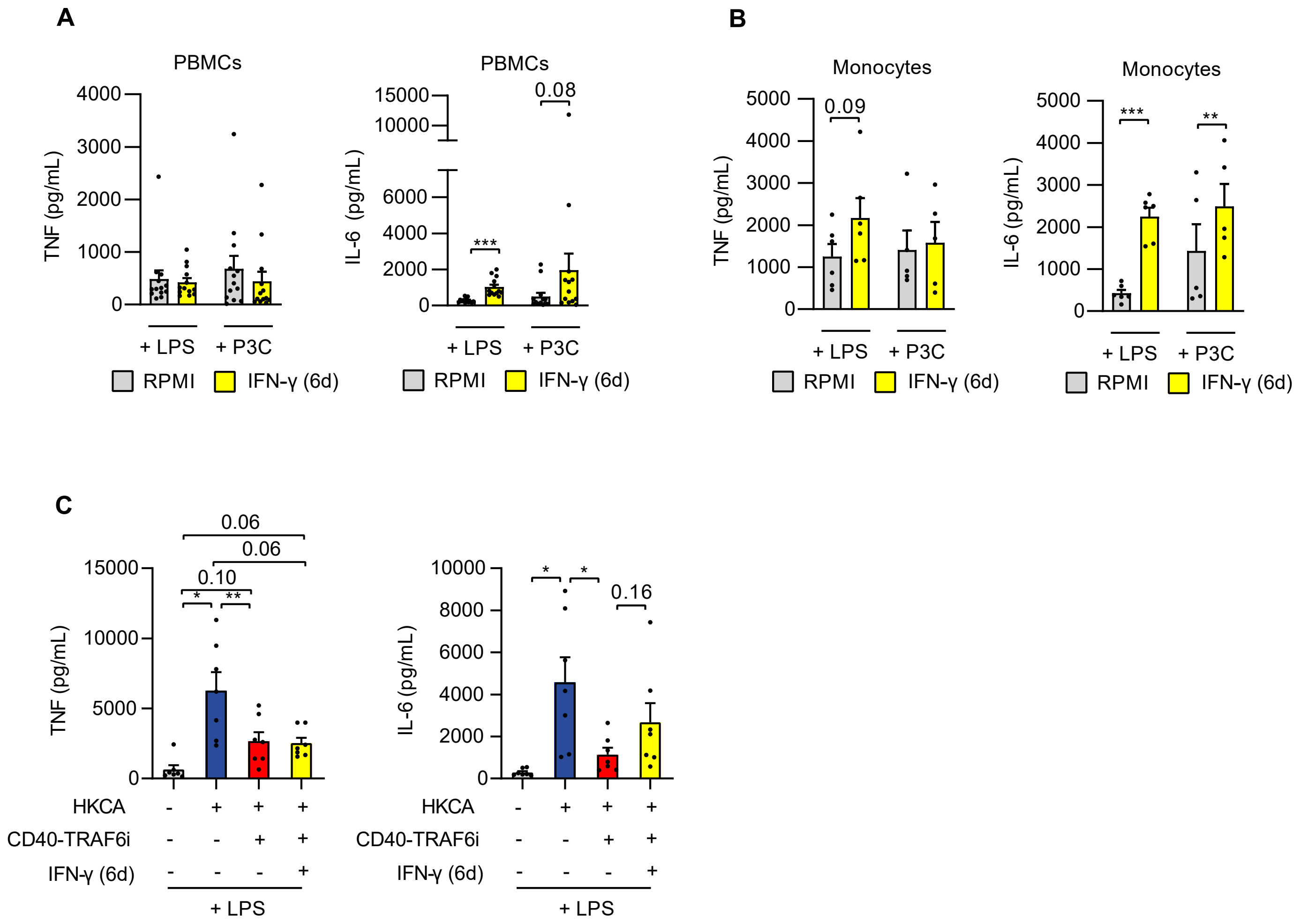
A
PBMCs
TNF (pg/mL)
4000
3000
2000
1000
0
+ LPS
+ P3C
RPMI
IFN-γ (6d)
PBMCs
IL-6 (pg/mL)
15000
10000
6000
4000
2000
0
***
0.08
+ LPS
+ P3C
RPMI
IFN-γ (6d)
B
Monocytes
TNF (pg/mL)
5000
4000
3000
2000
1000
0
0.09
+ LPS
+ P3C
RPMI
IFN-γ (6d)
Monocytes
IL-6 (pg/mL)
5000
4000
3000
2000
1000
0
***
**
+ LPS
+ P3C
RPMI
IFN-γ (6d)
C
TNF (pg/mL)
15000
10000
5000
0
0.06
0.06
0.10
*
**
HKCA - + + +
CD40-TRAF6i - - + +
IFN-γ (6d) - - - +
+ LPS
IL-6 (pg/mL)
10000
8000
6000
4000
2000
0
*
*
0.16
HKCA - + + +
CD40-TRAF6i - - + +
IFN-γ (6d) - - - +
+ LPS

Supplementary Figure 6: Role of IFN-γ priming in CD40-TRAF6i mediated inhibition of trained immunity responses in PBMCs. Related to Figure 2.

A: TNF and IL-6 production in adherent PBMCs treated for 6 days with IFN-γ or RPMI after restimulation with lipopolysaccharide (LPS) (n=13 donors) or Pam3CSK4 (P3C) (n=13 donors).

B: TNF and IL-6 production in purified monocytes treated for 6 days with IFN-γ or RPMI after restimulation with LPS (n=6 donors) or P3C (n=5 donors).

C: TNF and IL-6 production in adherent PBMCs treated for 24 h with heat-killed *Candida albicans* (HKCA) or RPMI in presence or absence of CD40-TRAF6i, and treated for 6 days with IFN-γ or RPMI, upon restimulation with LPS 6 days after HKCA- or RPMI stimulation (n=6 donors).

Mean ± SEM are shown. * $p < 0.05$, ** $p < 0.01$, *** $p < 0.001$. Paired t tests (A-B) and paired One-way ANOVA with Tukey's post-test (C).

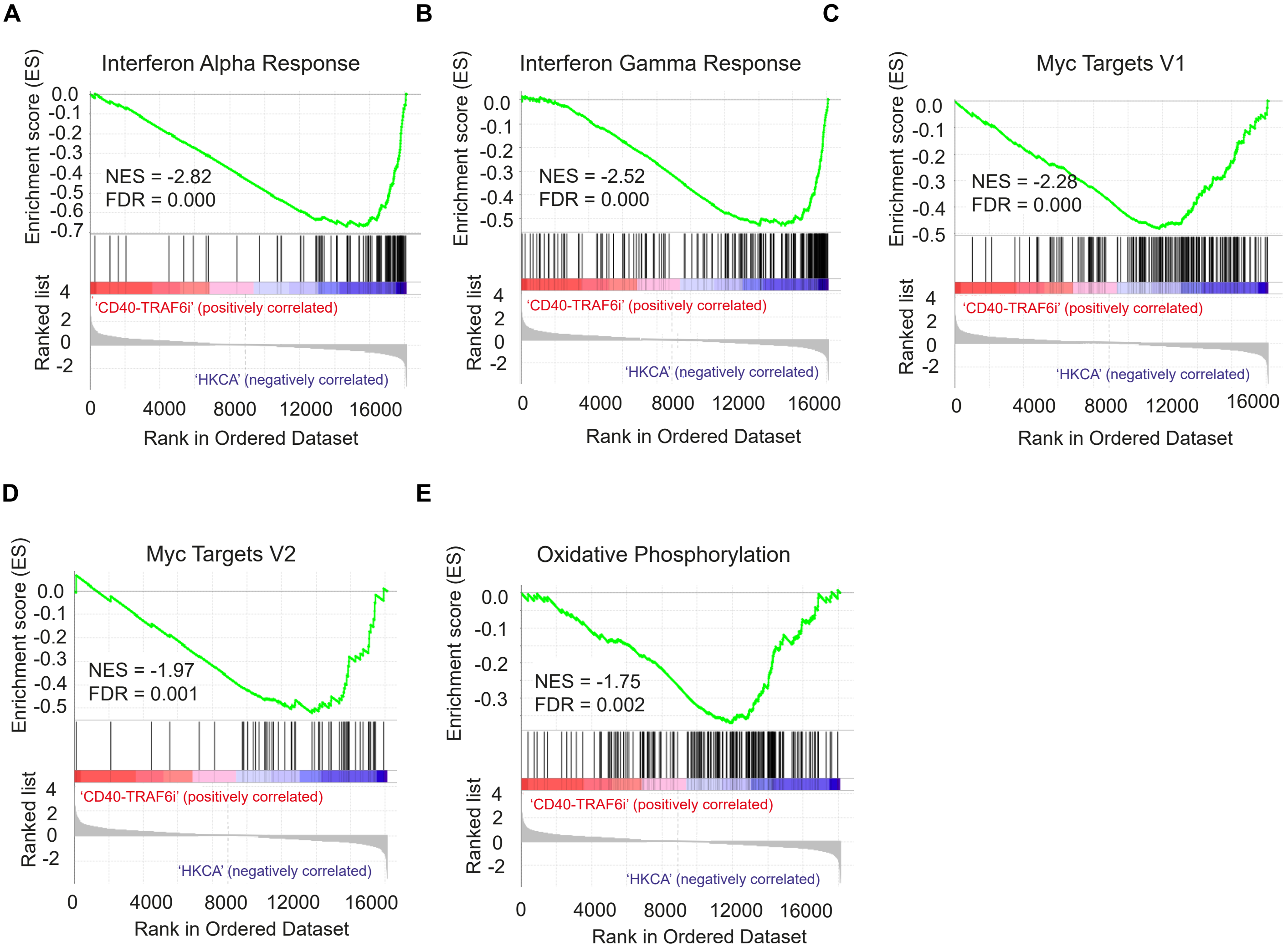

A
Interferon Alpha Response
Enrichment score (ES)
0.0
-0.1
-0.2
-0.3
-0.4
-0.5
-0.6
-0.7
NES = -2.82
FDR = 0.000
Ranked list
4
2
0
-2
'CD40-TRAF6i' (positively correlated)
'HKCA' (negatively correlated)
0
4000
8000
12000
16000
Rank in Ordered Dataset
B
Interferon Gamma Response
Enrichment score (ES)
0.0
-0.1
-0.2
-0.3
-0.4
-0.5
NES = -2.52
FDR = 0.000
Ranked list
4
2
0
-2
'CD40-TRAF6i' (positively correlated)
'HKCA' (negatively correlated)
0
4000
8000
12000
16000
Rank in Ordered Dataset
C
Myc Targets V1
Enrichment score (ES)
0.0
-0.1
-0.2
-0.3
-0.4
-0.5
NES = -2.28
FDR = 0.000
Ranked list
4
2
0
-2
'CD40-TRAF6i' (positively correlated)
'HKCA' (negatively correlated)
0
4000
8000
12000
16000
Rank in Ordered Dataset
D
Myc Targets V2
Enrichment score (ES)
0.0
-0.1
-0.2
-0.3
-0.4
-0.5
NES = -1.97
FDR = 0.001
Ranked list
4
2
0
-2
'CD40-TRAF6i' (positively correlated)
'HKCA' (negatively correlated)
0
4000
8000
12000
16000
Rank in Ordered Dataset
E
Oxidative Phosphorylation
Enrichment score (ES)
0.0
-0.1
-0.2
-0.3
NES = -1.75
FDR = 0.002
Ranked list
4
2
0
-2
'CD40-TRAF6i' (positively correlated)
'HKCA' (negatively correlated)
0
4000
8000
12000
16000
Rank in Ordered Dataset

Supplementary Figure 7: CD40-TRAF6 inhibition modulates transcriptomic changes induced by HKCA-training. Related to Figure 3.

A-E: Enrichment plots of HALLMARK gene sets ‘Interferon Alpha Response’ (A), ‘Interferon Gamma Response’ (B), ‘Myc Targets V1’ (C), ‘Myc Targets V2’ (D), and ‘Oxidative Phosphorylation’ (E) in monocytes treated for 24 hours with heat-killed *Candida albicans* (HKCA) in the presence of CD40-TRAF6i compared to monocytes treated with HKCA alone (n=3 donors per group).

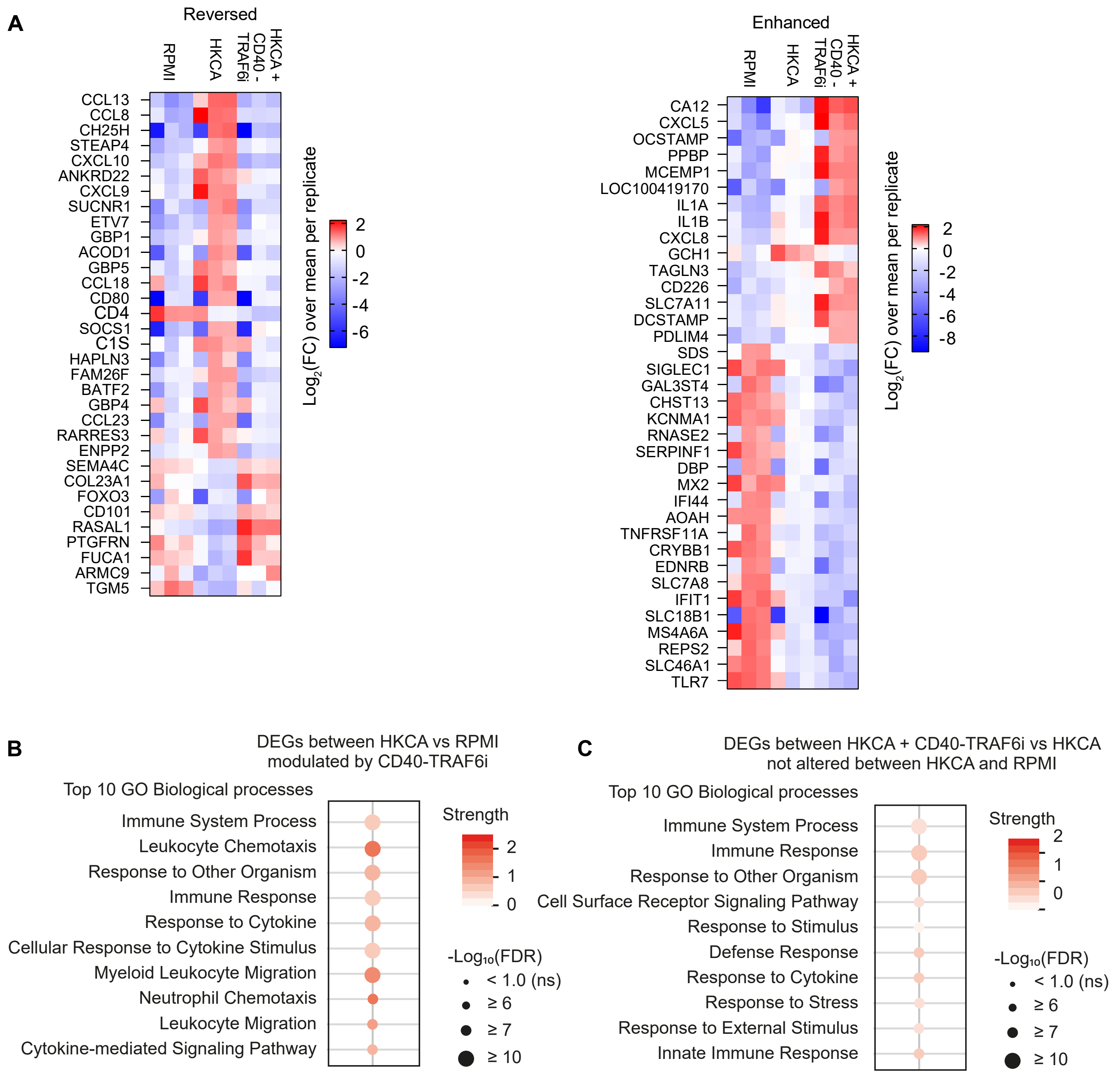
A
Reversed
RPMI
HKCA
HKCA + CD40 - TRAF6i
CCL13
CCL8
CH25H
STEAP4
CXCL10
ANKRD22
CXCL9
SUCNR1
ETV7
GBP1
ACOD1
GBP5
CCL18
CD80
CD4
SOCS1
C1S
HAPLN3
FAM26F
BATF2
GBP4
CCL23
RARRES3
ENPP2
SEMA4C
COL23A1
FOXO3
CD101
RASAL1
PTGFRN
FUCA1
ARMC9
TGM5
$Log_2(FC)$ over mean per replicate
2
0
-2
-4
-6
Enhanced
RPMI
HKCA
HKCA + CD40 - TRAF6i
CA12
CXCL5
OCSTAMP
PPBP
MCEMP1
LOC100419170
IL1A
IL1B
CXCL8
GCH1
TAGLN3
CD226
SLC7A11
DCSTAMP
PDLIM4
SDS
SIGLEC1
GAL3ST4
CHST13
KCNMA1
RNASE2
SERPINF1
DBP
MX2
IFI44
AOAH
TNFRSF11A
CRYBB1
EDNRB
SLC7A8
IFIT1
SLC18B1
MS4A6A
REPS2
SLC46A1
TLR7
$Log_2(FC)$ over mean per replicate
2
0
-2
-4
-6
-8
B
DEGs between HKCA vs RPMI modulated by CD40-TRAF6i
Top 10 GO Biological processes
Immune System Process
Leukocyte Chemotaxis
Response to Other Organism
Immune Response
Response to Cytokine
Cellular Response to Cytokine Stimulus
Myeloid Leukocyte Migration
Neutrophil Chemotaxis
Leukocyte Migration
Cytokine-mediated Signaling Pathway
Strength
2
1
0
$-Log_{10}(FDR)$
< 1.0 (ns)
≥ 6
≥ 7
≥ 10
C
DEGs between HKCA + CD40-TRAF6i vs HKCA not altered between HKCA and RPMI
Top 10 GO Biological processes
Immune System Process
Immune Response
Response to Other Organism
Cell Surface Receptor Signaling Pathway
Response to Stimulus
Defense Response
Response to Cytokine
Response to Stress
Response to External Stimulus
Innate Immune Response
Strength
2
1
0
$-Log_{10}(FDR)$
< 1.0 (ns)
≥ 6
≥ 7
≥ 10

Supplementary Figure 8: CD40-TRAF6 inhibition modulates transcriptomic changes induced by HKCA-training. Related to Figure 3.

A: Heat maps of 69 differentially expressed genes (DEGs) (Fold Change (FC) > 2 or < 0.5, False Discovery Rate (FDR) < 0.1) upon treatment with heat-killed *Candida albicans* (HKCA) compared to controls (RPMI), of which expression is significantly reduced (left) or enhanced (right) by CD40-TRAF6 inhibition, as determined with DESeq2 analysis (n=3 donors).

B: Top 10 enriched GO biological processes among 69 DEGs in HKCA-treated monocytes compared to RPMI-treated monocytes of which expression is significantly modulated (either reduced or enhanced) upon CD40-TRAF6i treatment, sorted on FDR (n=3 donors). (FC > 2 or < 0.5, FDR < 0.1)

C: Top 10 enriched GO biological processes among 261 DEGs in HKCA+CD40-TRAF6i-treated monocytes compared to HKCA-treated monocytes, that are not significantly different between HKCA-treated monocytes and untreated monocytes (RPMI), sorted on FDR (n=3 donors). (FC > 2 or < 0.5, FDR < 0.1).

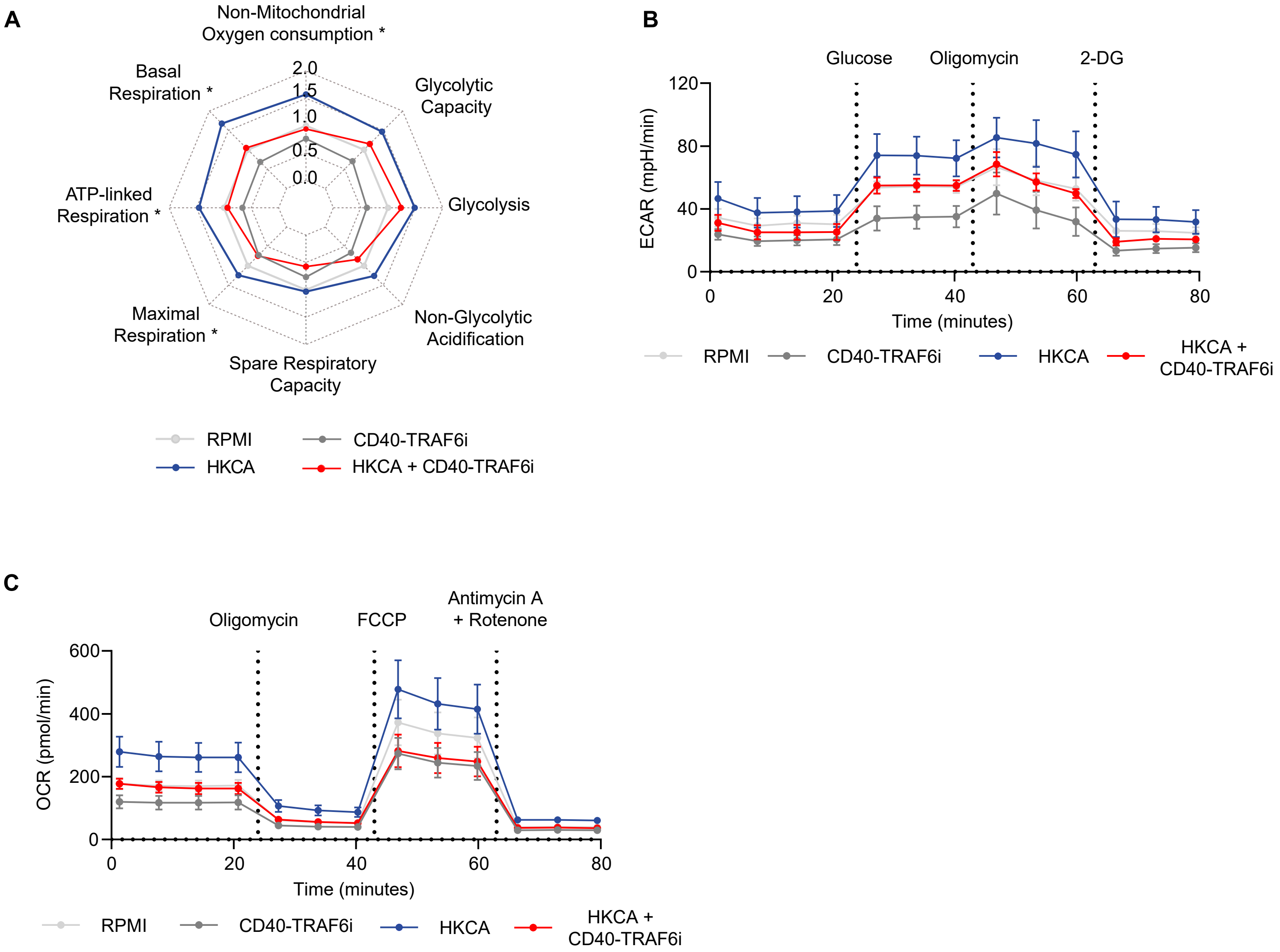
A
Non-Mitochondrial
Oxygen consumption *
Glycolytic
Capacity
Glycolysis
Non-Glycolytic
Acidification
Spare Respiratory
Capacity
Maximal
Respiration *
ATP-linked
Respiration *
Basal
Respiration *
2.0
1.5
1.0
0.5
0.0
RPMI
CD40-TRAF6i
HKCA
HKCA + CD40-TRAF6i
B
Glucose
Oligomycin
2-DG
ECAR (mpH/min)
Time (minutes)
RPMI
CD40-TRAF6i
HKCA
HKCA +
CD40-TRAF6i
C
Oligomycin
FCCP
Antimycin A
+ Rotenone
OCR (pmol/min)
Time (minutes)
RPMI
CD40-TRAF6i
HKCA
HKCA +
CD40-TRAF6i

Supplementary Figure 9: CD40-TRAF6 inhibition alters glycolysis and oxidative phosphorylation in HKCA-trained monocytes. Related to Figure 4.

A: Spider plot showing metabolic parameters 6 days after stimulation in monocytes isolated from PBMCs that were not treated or heat-killed *Candida albicans* (HKCA)-treated in the presence or absence of CD40-TRAF6i, for 24h (n=3-4 donors). Values were normalized to untrained monocytes. * indicates significant difference between HKCA+CD40-TRAF6i versus HKCA-treated monocytes as determined by one-sided paired t tests.

B: Extracellular acidification rate (ECAR) upon injection of glucose, oligomycin and 2-deoxyglucose (2-DG) at indicated time points, in monocytes purified from PBMCs that were not treated or HKCA-treated, in the presence or absence of CD40-TRAF6i, 6 days after treatment using Seahorse technology (n=3 donors).

C: Oxygen consumption rate (OCR) upon injection of oligomycin, Carbonyl cyanide-4-(trifluoromethoxy)phenylhydrazone) (FCCP) and antimycin A + rotenone at indicated time points, in monocytes purified from PBMCs that were not treated or HKCA-treated, in the presence or absence of CD40-TRAF6i, 6 days after treatment using Seahorse technology (n=4 donors).

Mean ± SEM are shown.

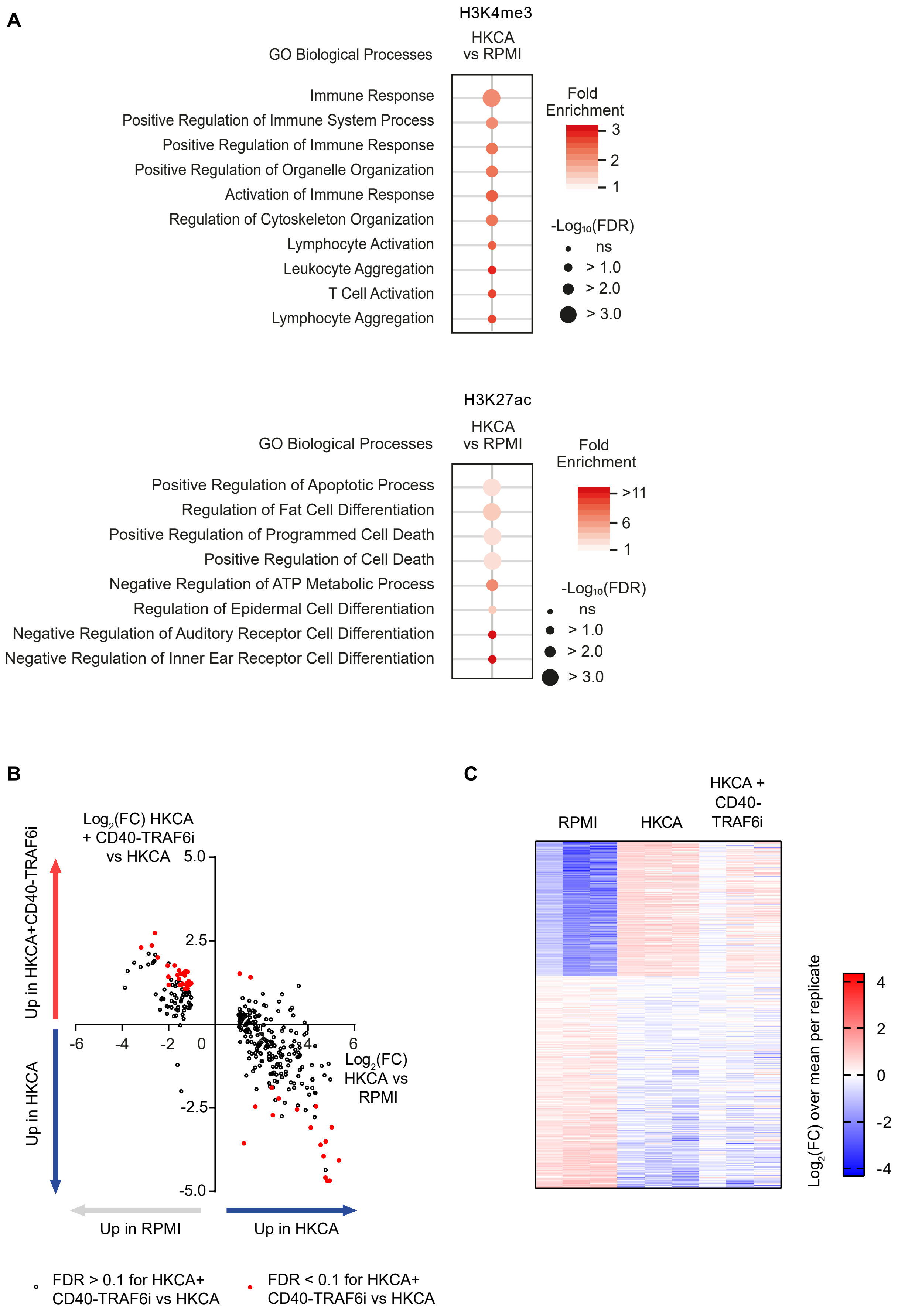
A
H3K4me3
HKCA vs RPMI
GO Biological Processes
Immune Response
Positive Regulation of Immune System Process
Positive Regulation of Immune Response
Positive Regulation of Organelle Organization
Activation of Immune Response
Regulation of Cytoskeleton Organization
Lymphocyte Activation
Leukocyte Aggregation
T Cell Activation
Lymphocyte Aggregation
Fold Enrichment
3
2
1
-Log10(FDR)
ns
> 1.0
> 2.0
> 3.0
H3K27ac
HKCA vs RPMI
GO Biological Processes
Positive Regulation of Apoptotic Process
Regulation of Fat Cell Differentiation
Positive Regulation of Programmed Cell Death
Positive Regulation of Cell Death
Negative Regulation of ATP Metabolic Process
Regulation of Epidermal Cell Differentiation
Negative Regulation of Auditory Receptor Cell Differentiation
Negative Regulation of Inner Ear Receptor Cell Differentiation
Fold Enrichment
>11
6
1
-Log10(FDR)
ns
> 1.0
> 2.0
> 3.0
B
Log2(FC) HKCA + CD40-TRAF6i vs HKCA
5.0
2.5
-2.5
-5.0
-6
-4
-2
0
4
6
Log2(FC) HKCA vs RPMI
Up in HKCA+CD40-TRAF6i
Up in HKCA
Up in RPMI
Up in HKCA
FDR > 0.1 for HKCA+ CD40-TRAF6i vs HKCA
FDR < 0.1 for HKCA+ CD40-TRAF6i vs HKCA
C
RPMI
HKCA
HKCA + CD40-TRAF6i
Log2(FC) over mean per replicate
4
2
0
-2
-4

Supplementary Figure 10: Effect of CD40-TRAF6 inhibition on H3K4me3 and H3K27ac modifications of HKCA-trained monocytes. Related to Figure 5.

A: Top 10 Gene Ontology (GO) Biological Processes associated with genomic regions showing altered H3K4me3 and H3K27ac in heat-killed *Candida albicans* (HKCA)-stimulated versus unstimulated monocytes (RPMI) (FC > 2 or < 0.5, FDR < 0.1) 6 days post-stimulation, determined using Genomic Regions Enrichment of Annotations Tool (n=3 donors).

B: Scatter plot showing $\log_2$-transformed FC for HKCA-treated monocytes versus untreated monocytes (RPMI) (x-axis) and HKCA+CD40-TRAF6i-treated monocytes versus HKCA-treated monocytes (y-axis) for 318 genomic regions with significantly altered H3K4me3 peak intensity in HKCA-treated versus RPMI-treated monocytes (FC > 2 or < 0.5, FDR < 0.1) determined with DESeq2. Red dots indicate regions in which H3K4me3 peak intensity was significantly different between HKCA+CD40-TRAF6i trained monocytes compared to HKCA-trained monocytes (FC > 2 or < 0.5, FDR <0.1).

C: Heat map showing the intensity of H3K27ac peaks in controls and monocytes stimulated with HKCA in the presence or absence of CD40-TRAF6 inhibitor, for 405 genomic regions with significantly altered H3K27ac peak intensity in HKCA-stimulated monocytes compared to controls, 6 days post-stimulation (fold change (FC) > 2 or < 0.5, false discovery rate (FDR) < 0.1) (n=3 donors).

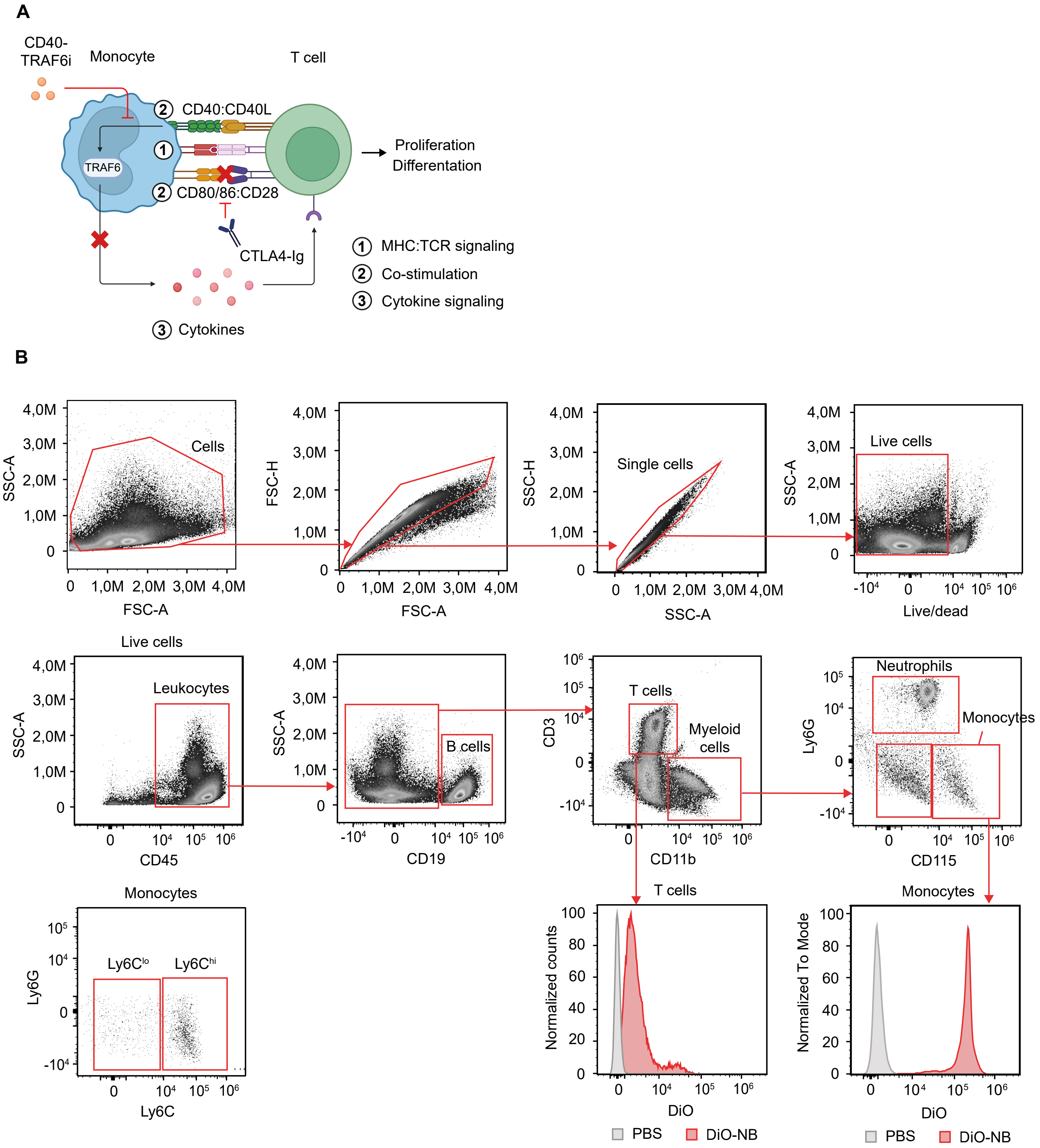
A
CD40-
TRAF6i
Monocyte
T cell
CD40:CD40L
TRAF6
CD80/86:CD28
CTLA4-Ig
Proliferation
Differentation
1 MHC:TCR signaling
2 Co-stimulation
3 Cytokine signaling
3 Cytokines
B
Cells
SSC-A
FSC-A
FSC-H
Single cells
Live cells
Live/dead
Leukocytes
CD45
B cells
CD19
T cells
Myeloid
cells
CD3
CD11b
Neutrophils
Monocytes
Ly6G
CD115
Ly6C^lo
Ly6C^hi
Ly6C
Normalized counts
Normalized To Mode
DiO
PBS
DiO-NB

Supplementary Figure 11: Hypothesized mechanisms of action of CD40-TRAF6i-nanobiologics and flow cytometric analysis for studying nanobiologic distribution *in vivo.* Related to Figure 7.

A: Schematic representation showing the hypothesized molecular effects of combined CTLA4-Ig and CD40-TRAF6i nanobiologic (CD40-TRAF6i-NB) treatment on the monocyte-induced activation of T cells.

B: Gating strategy for the quantification of DiO-nanobiologics (DiO-NBs) uptake in leukocyte populations in bone marrow, spleens, and grafts of C57BL/6J mice heterotopically transplanted with BALB/c hearts.

A

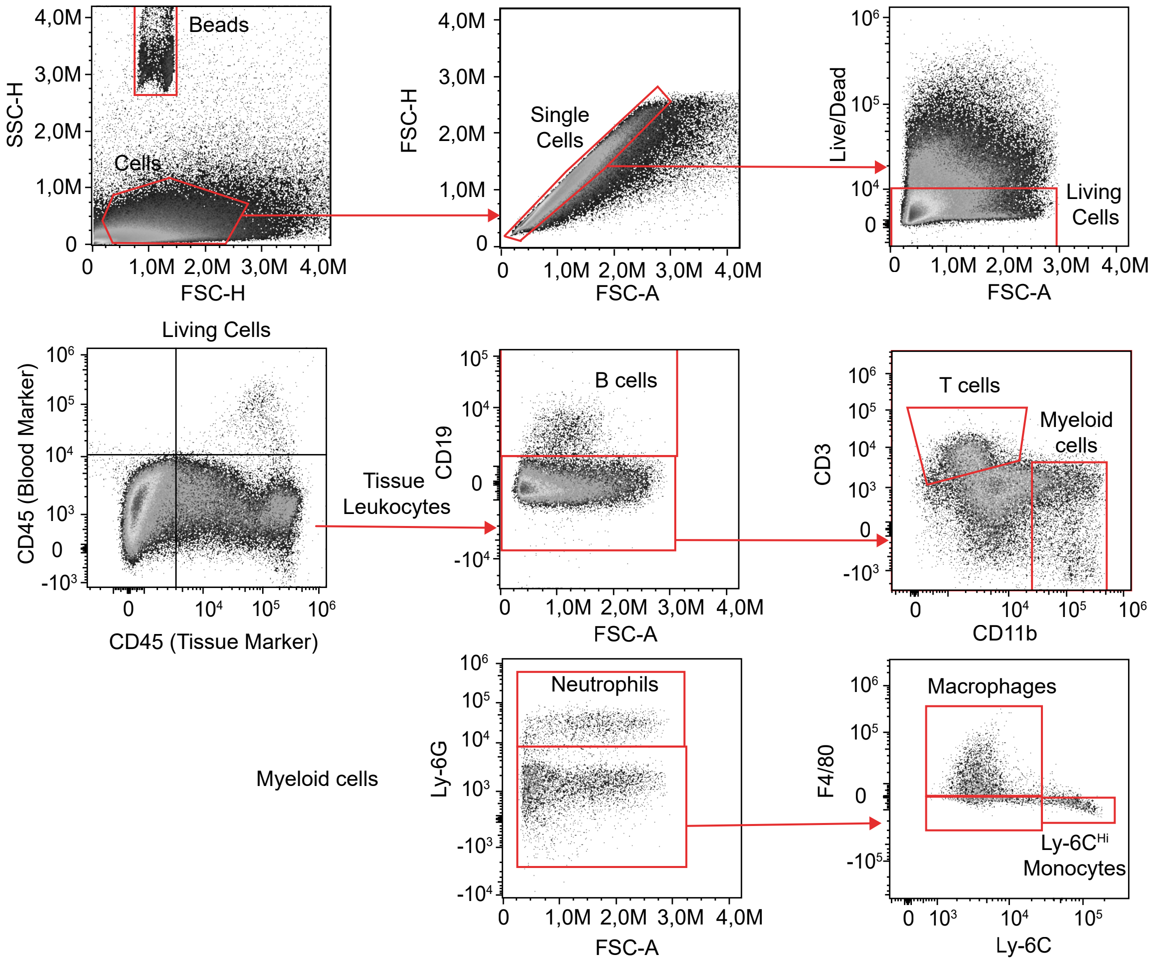

Beads
Cells
SSC-H
FSC-H
Single Cells
FSC-A
Live/Dead
Living Cells
CD45 (Blood Marker)
CD45 (Tissue Marker)
Tissue Leukocytes
CD19
B cells
T cells
Myeloid cells
CD3
CD11b
Myeloid cells
Neutrophils
Ly-6G
Macrophages
F4/80
Ly-6CHi Monocytes
Ly-6C

Supplementary Figure 12: Flow cytometric analysis of leukocytes in native hearts and allografts 100 days after heterotopic heart transplantation. Related to Figure 7.

A: Gating strategy for the quantification of leukocyte populations in native hearts and allografts 100 days after heterotopic heart transplantation.

Supplementary Table 1: RNA expression of co-stimulatory genes in heat-killed *Candida albicans*-trained monocytes versus untrained controls presented as Fold Change (FC).

Related to Figure 2.

Gene name	Alternative names	Fold Change (FC)	Adjusted P-value
CD274	B7-H, B7-H1, B7H1, PD-L1, PDCD1LG1, PDL1	3.2907	1,22E-10*
CD40	Bp50, TNFRSF5, p50	3.1144	3,62E-10*
CD48	BCM1, BLAST, SLAMF2, hCD48, mCD48	1.1596	3,17E+00
CD70	CD27L, CD27LG, TNFSF7	ND	ND
CD80	B7-1, B7.1, CD28LG, CD28LG1	3.1478	3,60E-02*
CD86	B7-2, B7.2, CD28LG2	0.7589	1,20E-01
HAVCR1	CD365, HAVCR, HAVCR-1, KIM1, TIM-1, TIM1, TIMD1	ND	2,14E+00
ICOSLG	B7-H2, B7H2, B7RP-1, B7RP1, B7h, CD275, GL50, ICOS-L, ICOSL, KIAA0653	ND	ND
LGALS9	LGALS9A	1.2460	ND
NECTIN2	CD112, HVEB, PRR2, PVRL2, PVRR2	1.4079	5,00E-02
NECTIN3	CD113, CDw113, DKFZP566B0846, PPR3, PVRL3, PVRR3, nectin-3	ND	ND
PDCD1LG2	B7-DC, Btdc, CD273, PD-L2, PDL2, bA574F11.2	1.5657	1,28E+00
PVR	CD155, HVED, NECL5, Necl-5, PVS, Tage4	1.3646	9,96E-01
SLAMF1	CD150, SLAM	2.3726	2,20E+00
TIMD4	TIM4	ND	ND
TNFRFS14	ATAR, CD270, HVEA, HVEM, LIGHTR, TR2	0.9270	9,95E+00
TNFRSF8	CD30, D1S166E, KI-1	0.3257	1,40E-02*
TNFRSF9	4-1BB, CD137, ILA	0.8672	7,59E+00
TNFSF14	CD258, HVEM-L, LIGHT, LTg	1.7684	3,76E-04
TNFSF15	MGC129934, MGC129935, TL1, TL1A, VEGI, VEGI192A	1.9475	1,63E-03
TNFSF18	AITRL, TL6, hGITRL	ND	ND
TNFSF4	CD252, OX-40L, TXGP1, gp34	ND	ND
VSIR	B7-H5, B7H5, C10orf54, Dies1, GI24, PD-1H, SISP1, VISTA	ND	ND

* : Significantly different expressed genes according to Bonferroni correction of p-values (FC > 2 or FC < 0.5, Adjusted p < 0.05) for selected list of genes

ND: indicates that FC and p-value could not be calculated because of too low number of detected reads.
